# Identification of *IGF1*, *SLC4A4*, *WWOX*, and *SFMBT1* as Hypertension Susceptibility Genes in Han Chinese with a Genome-Wide Gene-Based Association Study

**DOI:** 10.1371/journal.pone.0032907

**Published:** 2012-03-29

**Authors:** Hsin-Chou Yang, Yu-Jen Liang, Jaw-Wen Chen, Kuang-Mao Chiang, Chia-Min Chung, Hung-Yun Ho, Chih-Tai Ting, Tsung-Hsien Lin, Sheng-Hsiung Sheu, Wei-Chuan Tsai, Jyh-Hong Chen, Hsin-Bang Leu, Wei-Hsian Yin, Ting-Yu Chiu, Ching-Iuan Chern, Shing-Jong Lin, Brian Tomlinson, Youling Guo, Pak C. Sham, Stacey S. Cherny, Tai Hing Lam, G. Neil Thomas, Wen-Harn Pan

**Affiliations:** 1 Institute of Statistical Science, Academia Sinica, Taipei, Taiwan; 2 Institute of Biomedical Sciences, Academia Sinica, Taipei, Taiwan; 3 Graduate Institute of Biomedical Electronics and Bioinformatics, National Taiwan University, Taipei, Taiwan; 4 National Yang-Ming University School of Medicine and Taipei Veterans General Hospital, Taipei, Taiwan; 5 School of Public Health, National Medical Defense Center, Taipei, Taiwan; 6 Cardiovascular Center, Taichung Veterans General Hospital, Taichung, Taiwan; 7 Department of Internal Medicine, Kaohsiung Medical University Hospital, Kaohsiung, Taiwan; 8 Department of Internal Medicine, College of Medicine, National Cheng Kung University, Tainan, Taiwan; 9 Division of Cardiology, Cheng-Hsin Rehabilitation Medical Center, Taipei, Taiwan; 10 Division of Cardiology, Min-Sheng General Hospital, Taoyuan, Taiwan; 11 Department of Medicine and Therapeutics, The Chinese University of Hong Kong, Hong Kong, China; 12 Department of Psychiatry, The University of Hong Kong, Hong Kong, China; 13 The State Key Laboratory of Brain and Cognitive Sciences, The University of Hong Kong, Hong Kong, China; 14 School of Public Health, The University of Hong Kong, Hong Kong, China; 15 Public Health, Epidemiology and Biostatistics, School of Health and Population Sciences, University of Birmingham, Birmingham, United Kingdom; 16 Division of Preventive Medicine and Health Services Research, Institute of Population Health Sciences, National Health Research Institutes, Miaoli, Taiwan; National Institutes of Health, United States of America

## Abstract

Hypertension is a complex disorder with high prevalence rates all over the world. We conducted the first genome-wide gene-based association scan for hypertension in a Han Chinese population. By analyzing genome-wide single-nucleotide-polymorphism data of 400 matched pairs of young-onset hypertensive patients and normotensive controls genotyped with the Illumina HumanHap550-Duo BeadChip, 100 susceptibility genes for hypertension were identified and also validated with permutation tests. Seventeen of the 100 genes exhibited differential allelic and expression distributions between patient and control groups. These genes provided a good molecular signature for classifying hypertensive patients and normotensive controls. Among the 17 genes, *IGF1*, *SLC4A4*, *WWOX*, and *SFMBT1* were not only identified by our gene-based association scan and gene expression analysis but were also replicated by a gene-based association analysis of the Hong Kong Hypertension Study. Moreover, cis-acting expression quantitative trait loci associated with the differentially expressed genes were found and linked to hypertension. *IGF1*, which encodes insulin-like growth factor 1, is associated with cardiovascular disorders, metabolic syndrome, decreased body weight/size, and changes of insulin levels in mice. *SLC4A4*, which encodes the electrogenic sodium bicarbonate cotransporter 1, is associated with decreased body weight/size and abnormal ion homeostasis in mice. *WWOX*, which encodes the WW domain-containing protein, is related to hypoglycemia and hyperphosphatemia. *SFMBT1*, which encodes the scm-like with four MBT domains protein 1, is a novel hypertension gene. *GRB14*, *TMEM56* and *KIAA1797* exhibited highly significant differential allelic and expressed distributions between hypertensive patients and normotensive controls. *GRB14* was also found relevant to blood pressure in a previous genetic association study in East Asian populations. *TMEM56* and *KIAA1797* may be specific to Taiwanese populations, because they were not validated by the two replication studies. Identification of these genes enriches the collection of hypertension susceptibility genes, thereby shedding light on the etiology of hypertension in Han Chinese populations.

## Introduction

Hypertension [OMIN #145500] is a common complex disorder that is associated with both environmental and genetic factors [1] and exhibits moderate to high estimates of heritability ranging from 20% to 60% [2]. The disease has important public health effects throughout the world. One of every four adults globally is hypertensive, and the number of adults with hypertension in 2025 is predicted to increase by about 60% to a total of 1.56 billion people [2]. In Taiwan, one-third of adult males and one-fifth of adult females have hypertension [3]. Hypertension is one of the most critical risk factors for cerebral hemorrhage and infarction, coronary heart disease, heart failure, and kidney disease. The combination of these diseases contributes to a considerable proportion of mortality in the world.

Identifying hypertension susceptibility genes is potentially useful for understanding the complex genetic mechanism. Screening high-risk individuals and preventing disease development by early lifestyle and pharmacological treatment can contribute to the reduction of hypertension complications and cardiovascular mortality. Efforts to identify hypertension susceptibility genes have been ongoing for several decades. Candidate gene linkage analyses and genome-wide linkage approaches have identified hypertension susceptibility genes such as *ECE* (1p36.1) [4], *AGT* (1q42-43) [5], [6], *AGTR1A* (3q21-q25) [7], *HYT1* (17q) [8], [9], *HYT2* (15q) [9], *HYT3* (2p25-p24) [10], *HYT4* (12p) [11], *HYT5* (20q), *HYT6* (5p) [12], *HYT7* (3p14.1-q12.3) [13], and *HYT8* (18q21) [14]. In recent years, many single nucleotide polymorphism (SNP) markers have been discovered and broadly applied to study the genetics of complex diseases [15]. Methods to map the genes of complex diseases have evolved from linkage approaches for rare Mendelian traits to genome-wide association studies (GWAS). Several GWAS have been conducted for blood pressure or hypertension in recent years [16], [17], [18], [19], [20], [21], [22], [23], [24], [25], [26], [27], [28], [29], [30], [31], [32], [33], [34]. Blood pressure and hypertension susceptibility genes identified using GWAS are summarized (**Table 1**). However, most studies identified genes with small-to-moderate effects, and the genetic variations identified so far collectively explain only a small fraction of the genetic variability attributed to essential hypertension. In addition, most studies were conducted with Caucasian samples. Previous Asian studies were carried out primarily in Japanese and Korean populations and the findings may or may not be applicable to Han Chinese populations.

**Table 1:** A summary table of blood pressure and hypertension susceptibility genes identified by using GWAS.

Ref	Population	Analysis (n = sample size)	Blood pressure or hypertension- associated genes
[19]	Multi-ethnicity	Single-stage: GWAS (n = 1,260, 1,233 and 1,327 individuals in different analyses)	*CCL20*, *WDR69*, *CDH13*, *LPP*
[20]	Asian ancestry – Japan	Stage 1: GWAS (n = 188 cases+1,054 controls); Stage 2: Replication (n = 752 cases+752 controls); Stage 3: Replication (n = 619 cases+1,406 controls)	*ADD2*, *KIAA0789*, *M6PR*
[22]	Amish Caucasian	Stage 1: GWAS (n = 542 Amish Caucasians); Stage 2: Replication (n = 1,367 Amish Caucasians and 5,804 non-Amish Caucasians)	*STK39*
[23]	European ancestry	Stage 1: GWAS (n = 34,433); Stage 2: Replication (n = 71,225 subjects of European ancestry and 12,889 subjects of Indian Asian ancestry)	*CYP17A1*, *CYP17A2*, *FGF5*, *SH2B3*, *MTHFR*, ***C10orf107*** a, *ZNF652*, *PLCD3*
[24]	European ancestry – Germany	Stage 1: GWAS (n = 1,017 Germany and 364 cases+596 controls); Stage 2: Replication (n = 1,551 Germany and 447 cases+1,119 controls); Stage 3: Replication (n = 1,097 Estonians and 596 cases+650 controls; 2,401 British cases+1,969 controls)	*CDH13*
[25]	Asian ancestry – Korea	Stage 1: GWAS (n = 8,842 Koreans); Stage 2: Replication (n = 7,861 Koreans)	*ATP2B1*
[26]	African ancestry – America	Stage 1: GWAS (n = 509 African American cases+508 controls); Stage 2: Replication (n = 366 West African cases+614 controls)	*SLC24A4*, *CACNA1H*
[27]	European ancestry	Stage 1: GWAS (n = 29,136 European descent subjects); Stage 2: Replication (n = 34,433 European descent subjects)	*ATP2B1*, *CYP17A1*, *PLEKHA7*, *SH2B3*, *CACNB2*, *CSK ∼ ULK3*, *TBX3 ∼ TBX5*, *ULK4*
[28]	Asian ancestry	Stage 1: GWAS (n = 19,608 East Asian ancestry subjects); Stage 2: Replication (n = 10,518 East Asian ancestry subjects)	*ST7L ∼ CAPZA1*, *FIGN ∼ * ***GRB14*** a, *ENPEP*, *NPR3*, *TBX3*, *ALDH2*
[29]	Asian ancestry – Taiwan	Stage 1: GWAS (n = 175 cases+175 controls); Stage 2: Replication (n = 833 cases+833 controls)	*LOC344371*, *MYADML*, *FAM98A*, *RASGRP3*, *IMPG1*
[30]	African ancestry – America	Stage 1: GWAS (n = 8,591 African Americans); Stage 2: Replication (n = 11,882 African Americans); Stage 3: Replication (n = 69,899 European Americans)	*GPR98 ∼ ARRDC3*, *C21orf91*, *SLC25A42*, *HLA-B*
[31]	European ancestry – Sweden	Stage 1: GWAS (n = 1,621 cases+1,699 controls); Stage 2: Replication (n = 19,845 cases+16,541 controls)	*UMOD*
[32]	European ancestry – Great Britain	Single-stage: GWAS (n = 2,000 cases+3,000 controls)	*GPR39*, *XRCC4*, *MYO6*, *ZFAT*, *MACROD2*
[33]	Asian ancestry – Korea	Stage 1: GWAS (n = 7,551 Koreans); Stage 2: Replication (n = 3,703 Koreans)	*AKAP13*
[34]	Asian ancestry – Japan	Stage 1: GWAS (n = 936 Japanese); Stage 2: Replication (n = 3,228 Japanese); Stage 3: Replication (n = 2,895 Japanese)	*CCBE1*, *MAP7*, *ZFP64*, *PCDH18*, *CDH2*, ***WWOX*** a

a Genes (marked in bold) which were also identified to show differential allelic distributions between patient and control groups by our GBAS.

**Figure 1:**
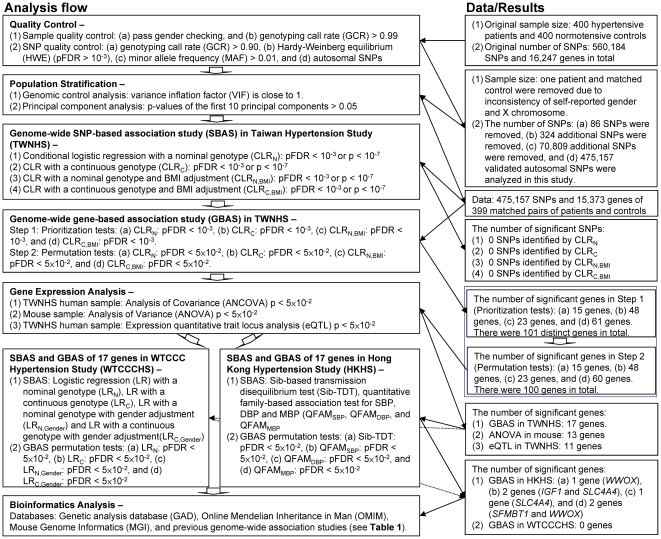
An analysis flowchart of this study.

In 2009, we published the first young-onset hypertension (YOH) genome-wide association mapping, which analyzed 175 patients with hypertension and 175 normotensive matched controls with the Affymetrix Human Mapping 100 K Set in the Han Chinese population of Taiwan [29]. We selected YOH as the phenotype of interest because it has a stronger genetic component than late-onset hypertension. Because we aimed to identify more disease genes responsible for YOH, we recruited more patient and control samples (400 YOH patients and 400 normotensive matched controls) and used a denser SNP genotyping platform (the Illumina HumanHap550-Duo BeadChip which provides 546,376 autosomal SNPs). There are 109 YOH patients and 98 normotensive controls used in both YOH GWAS. In addition to a genome-wide SNP-based association scan (genome-wide SBAS), based on an extension of our previously developed gene-based algorithms [35], we conducted a genome-wide gene-based association scan (genome-wide GBAS) to increase power, diminish the effect of locus heterogeneity, reduce the multiple testing burden, incorporate gene information, and provide a direct interpretation of association signals to genes. The genes identified with differential allelic associations in patient and control groups were then validated with gene expression analyses. We also studied expression quantitative trait loci (eQTL) associated with the differentially expressed genes identified in the present study and previous GWAS of hypertension [16], [17], [18], [19], [20], [21], [22], [23], [24], [25], [26], [27], [28], [29], [30], [31], [32], [33], [34] and linked them to hypertension. The analysis provides a deeper insight of phenotype-genotype relationship and illustrates that whether SNPs and genes influence YOH via gene regulation or via mechanisms rather than varied gene expression. Moreover, two replication analyses for this Taiwan Hypertension Study (TWNHS) were performed using genotype data from the Hong Kong Hypertension Study (HKHS) [36] and the Wellcome Trust Case-Control Consortium Hypertension Study (WTCCCHS) [17]. The HKHS collected samples from a Han Chinese population using a family-based study design, and the WTCCCHS collected samples from a Great Britain population using an independent case-control study design. A flowchart for the overall study design and analysis methods is provided in **Figure 1** and elaborated in the Materials and Methods section.

## Results

### Quality control and population stratification

For the 400 YOH patients and the 400 normotensive matched controls genotyped with the Illumina HumanHap550-Duo BeadChip, quality examination showed that all samples satisfied the genotype call rate (GCR) >0.99. One hypertensive patient and the matched normotensive control were excluded from the analysis due to an inconsistency of self-reported gender and X chromosome. Following quality examination of the SNPs, 86 SNPs with a GCR<0.9 were removed, and 324 additional SNPs were removed because of a departure from Hardy-Weinberg equilibrium (HWE) (pFDR<10^−3^) (where FDR is false discovery rate). An additional 70,809 SNPs were removed because of a minor allele frequency (MAF)<0.01. Ultimately, 475,157 validated SNPs (∼87% of autosomal SNPs on the HumanHap550-Duo BeadChip) were analyzed in this study. The impact of population substructures in our genome-wide SBAS and GBAS was evaluated. The variance inflation fraction (VIF) was 1.027, which is close to 1 and suggests that the population admixture with respect to association tests was not significant in this study. The principal component plot shows all hypertensive and normotensive samples randomly mixed together (**Figure S1**). Moreover, the results of non-parametric median tests also show no significant differences between patient and control groups on the first ten principal components (p-values for the first ten principal components were 0.435, 0.523, 0.620, 0.178, 0.177, 0.435, 0.435, 0.831, 0.055 and 0.435). The results illustrate no substructure in patient and control groups in this study.

### Genome-wide SBAS

Initially, genome-wide SBAS was performed with four conditional logistic regression models abbreviated “CLR_N_”, “CLR_C_”, “CLR_N,BMI_”, and “CLR_C,BMI_”, in which the subscript “N” stands for a nominal SNP coding for a genotype-based analysis, and the subscript “C” stands for a continuous SNP coding for a trend-based analysis without or with a covariate adjustment for body mass index (BMI). No significant SNPs (pFDR<10^−3^ or p<10^−7^) were found. However, we observed that some genomic regions contained a significant proportion of SNPs with marginal significance (**Figure S2A–S2D**). For example, the region in *KIAA1797* between 20,638,805 bp and 20,993,372 bp on chromosome 9p21 contains 84 SNPs probed on the HumanHap550−Duo BeadChip. The proportions of SNPs with marginal significance (p<5×10^−2^) in the four conditional logistic regression analyses (CLR_N_, CLR_C_, CLR_N,BMI_, and CLR_C,BMI_) were 10%, 26%, 20%, and 23%, with the smallest values of p-values on *KIAA1797* of 7.56×10^−4^, 7.38×10^−4^, 4.31×10^−5^, and 7.69×10^−6^, respectively. P-values of genome-wide SBAS can be downloaded at the website, http://pan.ibms.sinica.edu.tw/hypertension/data.

### Genome-wide GBAS

**Figure 2:**
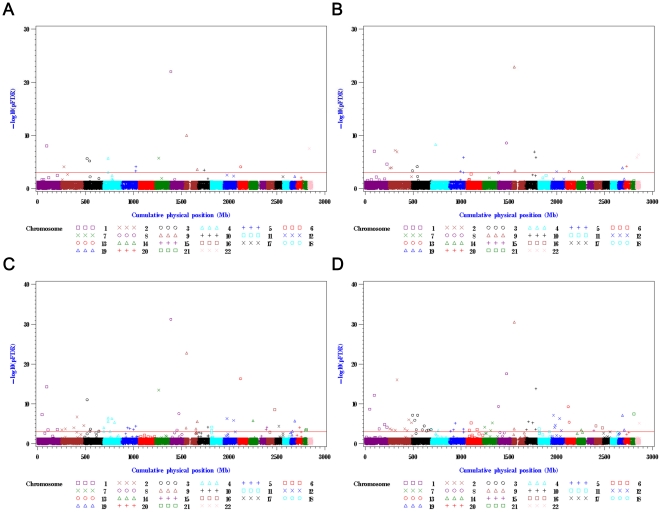
Gene-based association tests based on p-values from the four conditional logistic regression models. (A) model CLR_N_, (B) model CLR_N,BMI_, (C) model CLR_C_, and (D) model CLR_C,BMI_. In each figure, the vertical axis is the FDR-adjusted p-values (−log_10_ scale) of gene-based association tests, and the horizontal axis is cumulative physical position (Mb scale). The red reference line signifies pFDR = 10^−3^.

Genome-wide GBAS was then applied to increase the statistical power in the context of gene regions. The method of genome-wide GBAS is introduced in **Method S1**. The analysis identified 101 potential hypertension susceptibility genes (pFDR<10^−3^) in at least one of the four genome-wide GBAS models, which combined p-values obtained from the genome-wide SBAS (**Figure 2A–2D** **; Table S1**). The 101 genes are classified into 12 categories (Category I to XII) according to gene significance in the four genome-wide GBAS models. In **Table S1**, three genes were identified by all four genome-wide GBAS regression models (Category I), six genes were identified by three of the four models (Categories II, III and VII), 25 genes were identified by two of the four models (Categories IV, V, VIII and X), and 67 genes were identified by only one model (Categories VI, IX, XI and XII). Empirical p-values of the 101 genes were calculated using a permutation procedure with 10,000 replications. The gene-trait association information collected from the Genetic Association Database (GAD), Online Mendelian Inheritance in Man (OMIM), and Mouse Genome Informatics (MGI) databases for the 101 genes is summarized in **Table S2**. All the genes (except for *FMO5*) were successfully validated (pFDR<5×10^−2^).

### Gene expression analysis

**Figure 3:**
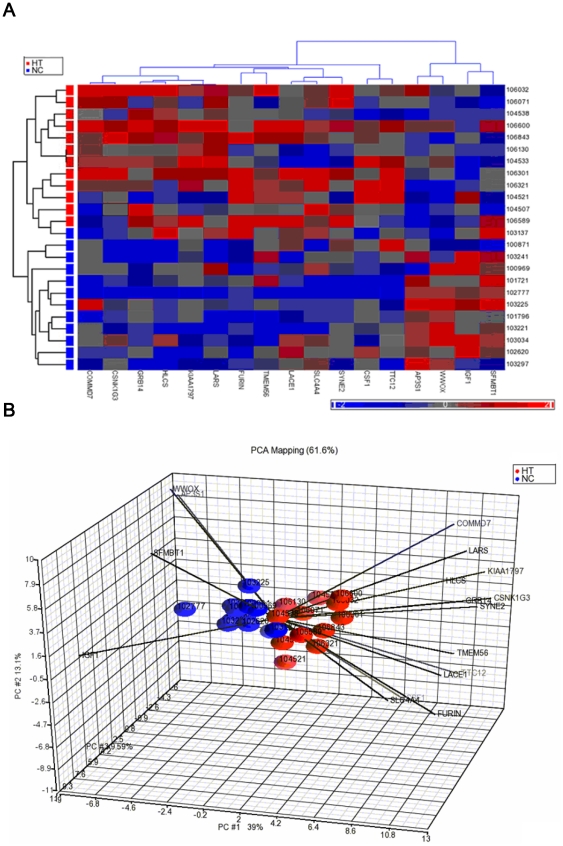
Evaluation of the 17 differentially expressed genes in hypertension. (A) Cluster analysis of transcriptional data from 12 pairs of patients with hypertension (HT) and normotensive controls (NC). (B) Principal component analysis (PCA) of transcriptional data from 12 pairs of patients with hypertension and normotensive controls), where the variation explained by the first two principal components (PC #1 and PC #2) is 39% and 13.1%.

As a validation process, gene expression analysis was conducted to compare transcriptional levels of the 100 identified genes for 12 matched patient–control pairs in which the 12 matched pairs of patients and controls were selected to have an extremely discordant genotypic distribution between groups. The method for selecting extremely discordant case-control pairs is introduced in **Method S2**. Among the 100 genes, gene expression data for 92 genes were provided by the Illumina HT12 Expression BeadChip, of which 17 genes were differentially expressed (p<5×10^−2^) (**Table 2**). Beta coefficients and their standard errors from ANCOVA are also provided (**Table S3**). Normalized gene expression data of the 17 genes can be downloaded at the website, http://pan.ibms.sinica.edu.tw/hypertension/data. These 17 genes showed not only differential allelic distributions but also differential transcriptional distributions between patient and control groups. Based on the gene expression data of the 17 differentially expressed genes, a cluster analysis of 12 pairs of hypertensive patients and normotensive controls only misclassified one normotensive control to the patient group (**Figure 3A**). The first three principal components of expression of the 17 genes explained ∼62% of the total variation for the 12 matched pairs of YOH patients and normotensive controls (**Figure 3B**). Fifteen of the 17 genes had data of gene expression in mouse. Tissue-specific analysis of gene expression showed that, except for *LARS* and *FURIN*, other genes exhibited differential transcriptional distributions between genetically hypertensive and normotensive inbred mouse strains in at least one tissue (p<5×10^−2^) (**Table 2**). Beta coefficients and their standard errors from ANOVA are also provided (**Table S3**).

**Table 2:** Seventeen genes with differential allelic and transcriptional distributions identified by the TWNHS.

Gene	Raw p-values of GBAS of conditional logistic model^a^^,^ b				Raw p-values of the human and mouse GE analysis^a^^,^ b				
	CLR_N_	CLR_C_	CLR_N,BMI_	CLR_C,BMI_	Human	Mouse aorta	Mouse heart	Mouse kidney	Mouse liver
*TMEM56*	**9.83E-14**	**6.78E-20**	**1.74E-12**	**1.19E-17**	**5.77E-03**	**3.81E-03**	**4.48E-02**	5.31E-02	**1.39E-02**
*KIAA1797*	**6.78E-16**	**1.13E-28**	**4.78E-29**	**1.03E-36**	**7.97E-03**	NA^c^	NA^c^	NA^c^	NA^c^
*FMBT1*	**1.82E-10**	6.93E-01	**3.59E-09**	6.93E-01	**4.74E-02**	**7.53E-03**	7.18E-01	1.00E-01	6.60E-01
*LARS*	**2.80E-09**	1.44E-05	**5.28E-11**	2.24E-05	**6.83E-03**	8.54E-01	7.77E-02	6.03E-01	1.38E-01
*GRB14*	2.56E-04	**7.04E-12**	8.64E-04	**2.50E-08**	**7.15E-03**	5.04E-01	**3.79E-02**	**3.57E-02**	2.21E-01
*IGF1*	**3.10E-07**	**6.97E-11**	3.10E-04	**9.90E-08**	**3.21E-03**	**3.29E-02**	5.70E-02	1.09E-01	1.71E-01
*FURIN*	**3.62E-05**	**3.34E-08**	**4.76E-05**	**8.66E-08**	**2.26E-03**	4.96E-01	1.72E-01	6.05E-01	6.51E-01
*WWOX*	1.70E-02	**7.47E-14**	2.23E-02	**1.18E-08**	**5.96E-03**	**2.19E-02**	9.94E-02	2.37E-01	**4.22E-03**
*HLCS*	**5.88E-05**	**3.51E-08**	**5.15E-06**	**1.09E-12**	**1.38E-02**	**4.02E-02**	**1.28E-02**	1.66E-01	**1.90E-03**
*CSF1*	**5.66E-05**	**4.67E-08**	8.36E-02	**9.98E-06**	**2.76E-02**	**6.44E-03**	7.60E-01	**1.54E-02**	3.20E-01
*CSNK1G3*	3.23E-02	**3.39E-08**	3.82E-01	1.06E-01	**2.71E-03**	**1.10E-03**	**4.99E-03**	**2.85E-05**	1.00E-01
*COMMD7*	**2.50E-04**	**8.74E-09**	1.18E-01	**6.81E-04**	**2.62E-02**	**4.48E-02**	6.55E-01	5.31E-01	7.99E-01
*AP3S1*	1.38E-02	5.17E-02	**3.92E-08**	3.22E-02	**8.03E-03**	NA^c^	NA^c^	NA^c^	NA^c^
*LC4A4*	7.58E-01	1.90E-02	**3.81E-05**	**7.26E-08**	**3.14E-02**	**3.32E-04**	**1.62E-03**	**4.42E-02**	2.69E-01
*LACE1*	5.37E-01	4.82E-01	1.46E-03	**4.24E-08**	**1.86E-02**	**4.14E-03**	6.17E-01	2.96E-01	5.80E-01
*TTC12*	1.85E-03	1.01E-03	1.23E-02	**2.03E-07**	**2.33E-02**	**4.69E-02**	**5.78E-07**	2.87E-01	6.00E-01
*SYNE2*	9.62E-01	6.71E-01	2.63E-01	**4.52E-08**	**1.61E-02**	1.55E-01	4.71E-01	**2.48E-02**	**2.18E-02**

a Test results include p-values of the gene-based association analysis (CLR_N_, CLR_C_, CLR_N,BMI_, and CLR_C,BMI_) and −log_10_(p) of gene expression [GE] analysis.

b Raw p-values of genes that reached pFDR<0.05 in GBAS of the TWNHS and p-values of genes that reached p<0.05 in gene expression studies are marked in bold.

c NA denotes that expression data of a gene were not available in the mouse gene expression analysis.

We made use of the Genevar database version 3.0.1 [37] to investigate cis-acting expression quantitative trait loci (cis-eQTL) associated with the 17 differentially expressed genes identified in our human gene expression study (**Table 2**). Among the 17 genes, only *AP3S1* did not contain cis-eQTL. The gene-centric cis-eQTL analysis found 361 cis-eQTL (p<5×10^−2^) in 2-Mb windows around the 16 genes, where 285 cis-eQTL on 13 genes were interrogated in the Illumina HumanHap550-Duo BeadChip. There were also 70 cis-eQTL on 11 genes (*TMEM56*, *KIAA1797*, *LARS*, *GRB14*, *IGF1*, *WWOX*, *HLCS*, *CSF1*, *CSNK1G3*, *TTC12* and *SYNE2*) associated with YOH in our TWNHS study (p<5×10^−2^), where the disease associated alleles of 40 cis-eQTL increased genetic risk of YOH as well as up-regulated gene expression (**Table S4**). In addition to the 17 genes in **Table 2**, we also examined cis-eQTL of the hypertension-associated genes identified by previous hypertension genomic studies [16], [17], [18], [19], [20], [21], [22], [23], [24], [25], [26], [27], [28], [29], [30], [31], [32], [33], [34] in **Table 1** where *GRB14* and *WWOX* were excluded here because they were also identified by the present study and their cis-eQTL were already examined. Cis-eQTL of 54 genes were studied, although 6 of these genes (*CYP17A2*, *HLA-B*, *KIAA0789*, *LOC344371*, *MYADML* and *ULK4*) did not have cis-eQTL in Genevar database [37]. The gene-centric cis-eQTL analysis found 1,902 cis-eQTL (p<5×10^−2^) in 2-Mb windows around the 48 genes, where 1,628 cis-eQTL on 33 genes were interrogated in the Illumina HumanHap550-Duo BeadChip. Finally, 31 cis-eQTL on 9 genes (*AKAP13*, *CACNB2*, *CCBE1*, *CDH13*, *CSK*, *FIGN*, *LPP*, *SLC24A4* and *TBX3*) were associated with YOH in our TWNHS study (p<5×10^−2^), where the disease associated alleles of 9 cis-eQTL increased genetic risk of YOH as well as up-regulated gene expression (**Table S5**).

### Replication analysis

In the HKHS replication analysis, genome-wide SBAS contained “sib-TDT”, “QFAM_SBP_”, “QFAM_DBP_”, and “QFAM_MBP_” for analyzing dichotomous disease status and three quantitative traits (quantitative systolic blood pressure [SBP], quantitative diastolic blood pressure [DBP], and quantitative mean blood pressure [MBP]). In the WTCCCHS, genome-wide SBAS was performed with four logistic regression models abbreviated “LR_N_”, “LR_C_”, “LR_N,Gender_”, and “LR_C,Gender_” in which the subscript “N” stands for a nominal SNP coding for a genotype-based analysis, and the subscript “C” stands for a continuous SNP coding for a trend-based analysis with or without a covariate adjustment for gender. Among the 17 genes that exhibited differential allelic distributions and differential transcriptional distributions between patient and control groups, four hypertension-associated genes (*IGF1*, *SLC4A4*, *WWOX*, and *SFMBT1*) were further confirmed by replication studies. The results are summarized in **Table 3**.

**Table 3:** Replication analysis of the seventeen genes with differential allelic and transcriptional distributions in cases and controls.

Gene	Raw p-values of GBAS of the HKHS^a^^,^ b				Raw p-values of GBAS of the WTCCCHS^a^^,^ b			
	sTDT	QFAM_SBP_	QFAM_DBP_	QFAM_MBP_	LR_N_	LR_C_	LR_N,Gender_	LR_C,Gender_
*TMEM56*	5.60E-01	5.60E-01	5.60E-01	5.60E-01	8.06E-01	7.15E-01	7.69E-01	6.61E-01
*KIAA1797*	9.75E-01	9.86E-01	4.84E-01	9.86E-01	7.99E-01	9.34E-01	6.58E-01	9.34E-01
*SFMBT1*	3.04E-01	6.93E-01	2.91E-01	**1.36E-05**	5.12E-01	4.83E-01	5.12E-01	5.12E-01
*LARS*	2.26E-01	2.26E-01	2.26E-01	2.26E-01	3.37E-01	3.37E-01	3.37E-01	3.27E-01
*GRB14*	6.93E-01	6.93E-01	6.93E-01	6.93E-01	8.58E-01	8.58E-01	8.58E-01	8.58E-01
*IGF1*	2.02E-01	**1.21E-04**	6.03E-01	6.03E-01	8.06E-01	7.46E-01	7.84E-01	8.51E-01
*FURIN*	2.26E-01	2.26E-01	2.26E-01	2.26E-01	5.00E-02	5.00E-02	5.00E-02	5.00E-02
*WWOX*	**1.05E-06**	1.11E-03	1.54E-02	**8.82E-07**	3.83E-01	8.68E-01	3.85E-01	9.35E-01
*HLCS*	6.93E-01	5.08E-01	9.29E-01	9.34E-01	8.06E-01	4.36E-01	7.64E-01	6.16E-01
*CSF1*	4.31E-01	4.31E-01	4.26E-01	3.88E-01	5.66E-02	1.98E-02	6.00E-02	2.15E-02
*CSNK1G3*	5.82E-01	5.82E-01	5.82E-01	5.82E-01	1.44E-02	3.22E-01	1.19E-02	3.21E-01
*COMMD7*	2.26E-01	2.26E-01	2.26E-01	2.26E-01	6.71E-02	1.52E-03	3.06E-01	1.11E-02
*AP3S1*	3.37E-01	3.37E-01	3.37E-01	3.37E-01	2.26E-01	2.26E-01	2.26E-01	2.26E-01
*SLC4A4*	4.33E-02	**9.19E-06**	**1.19E-03**	4.61E-03	9.43E-01	6.76E-01	9.43E-01	6.87E-01
*LACE1*	4.58E-01	5.37E-01	5.37E-01	5.37E-01	7.38E-02	4.89E-02	7.23E-02	4.73E-02
*TTC12*	6.03E-01	7.20E-03	6.03E-01	2.01E-01	6.42E-01	6.42E-01	6.42E-01	6.42E-01
*SYNE2*	9.08E-01	8.78E-01	8.83E-01	9.61E-01	9.54E-01	8.76E-01	9.54E-01	8.85E-01

a Test results include p-values of GBAS of the HKHS (Method: sTDT, QFAM_SBP_, QFAM_DBP_, and QFAM_MBP_) and GBAS of the WTCCCHS (Method: LC_N_, LC_C_, LC_N,Gender_, and LC_C,Gender_) of the 17 genes characterized by both differential allelic distributions and transcriptional distributions between patient and control groups.

b Raw p-values of genes that reached pFDR<5×10^−2^ in the GBAS of the HKHS and the WTCCCHS are marked in bold.

*IGF1*, which encodes insulin-like growth factor on 12q23.2, was not only identified by the GBAS in our TWNHS [p_CLRN_ = 3.10×10^−7^ (pFDR_CLRN_ = 2.47×10^−2^); p_CLRC_ = 6.97×10^−11^ (pFDR_CLRC_ = 2.20×10^−2^); p_CLRC,BMI_ = 9.90×10^−8^ (pFDR_CLRC,BMI_ = 2.95×10^−2^)] and gene expression analysis [p_ANCOVA_ = 3.21×10^−3^] but was also identified by a GBAS of the HKHS [p_QFAMSBP_ = 1.21×10^−4^ (pFDR_QFAMSBP_ = 3.73×10^−2^)]. Moreover, association between gene expression and cis-eQTL markers of *IGF1* was also found in our gene-centric cis-eQTL analysis (**Table S4**). Two cis-eQTL of *IGF1* were found (rs10860862 and rs5742632) where the latter SNP is an intronic enhancer at a transcriptional factor binding site. *IGF1* is a well-documented gene that plays a role in cardiovascular disorders [38], [39], [40], [41], [42] and metabolic disorders [43], [44], [45], [46], [47], [48], [49], [50], [51], [52]. *IGF1* variants are associated with decreased body weight/size, abnormal glucose homeostasis, increased/decreased circulating insulin levels, decreased circulating *IGF1* levels, and increased DBP/SBP values in mice [53], [54], [55].

*SLC4A4*, which encodes the electrogenic sodium bicarbonate cotransporter 1 on 4q21, was not only identified by our GBAS [p_CLRN,BMI_ = 3.81×10^−5^ (pFDR_CLRN,BMI_ = 2.99×10^−2^); p_CLRC,BMI_ = 7.26×10^−8^ (pFDR_CLRC,BMI_ = 2.64×10^−2^)] and gene expression analysis [p_ANCOVA_ = 3.14×10^−2^] but was also identified by a GBAS of the HKHS [p_QFAMSBP_ = 9.19×10^−6^ (pFDR_QFAMSBP_ = 1.63×10^−2^); p_QFAMDBP_ = 1.19×10^−3^ (pFDR_QFAMDBP_ = 4.79×10^−2^)]. This gene is associated with decreased body weight/size, hematocrit, and abnormal ion homeostasis in mice [56]. In addition, *SLC4A5* is a member of the same family of solute carriers as *SLC4* and was also shown to be associated with blood pressure [57] and with hypertension [58].

*WWOX*, which encodes the WW domain-containing protein on 16q23.3-q24.1, is the third gene that was simultaneously identified by our genome-wide GBAS [p_CLRC_ = 7.47×10^−14^ (pFDR_CLRC_ = 5.77×10^−3^) and p_CLRC,BMI_ = 1.18×10^−8^ (pFDR_CLRC,BMI_ = 1.84×10^−2^)], gene expression analysis [p_ANCOVA_ = 5.96×10^−3^], and a GBAS of the HKHS [p_sTDT_ = 1.05×10^−6^ (p_sTDT_ = 1.37×10^−2^) and p_QFAMMBP_ = 8.82×10^−7^ (p_QFAMMBP_ = 1.05×10^−2^)]. Moreover, association between gene expression and cis-eQTL markers of *WWOX* was also found in our gene-centric cis-eQTL analysis (**Table S4**). In total, 24 cis-eQTL were found, where 14 cis-eQTL are intronic enhancers at transcriptional factor binding sites, 1 cis-eQTL locates in *WWOX* upstream, and the remaining 9 cis-eQTL are in an intronic region with no known function. *WWOX* variants are associated with hypoglycemia, hyperphosphatemia, decreased circulating cholesterol levels and triglyceride levels, but with increased blood uric acid levels in mice [59]. *WWOX* variants were also found to be associated with blood pressure in a previous Japanese GWAS [23].

*SFMBT1*, which encodes the drosophila sex comb on middle (scm)-like protein on 3p21.31 and contains four malignant brain tumor repeat domains, was simultaneously identified by our genome-wide GBAS [p_CLRN_ = 1.82×10^−10^ (pFDR_CLRN_ = 2.47×10^−2^) and p_CLRN,BMI_ = 3.59×10^−9^ (pFDR_CLRN,BMI_ = 2.81×10^−2^)], gene expression analysis [p_ANCOVA_ = 4.74×10^−2^], and a GBAS of the HKHS [p_QFAMMBP_ = 1.36×10^−5^ (pFDR_QFAMMBP_ = 3.44×10^−2^)]. This gene may have a novel role in hypertension, and its role in disease etiology should be studied further.

## Discussion

We conducted the first genome-wide GBAS, using a novel region- and context-based method, to identify hypertension susceptibility genes in a Han Chinese population. Four hypertension susceptibility genes, *IGF1*, *SLC4A4*, *WWOX*, and *SFMBT1*, were found by a statistical permutation procedure, confirmed by gene expression analysis, and further replicated using data from the HKHS. Among them, *IGF1*, *SLC4A4*, and *WWOX* were previously implicated as hypertension candidate genes in humans and mice and are primarily associated with obesity, glucose metabolism, and ion homeostasis, which are well-known mechanisms of blood pressure regulation. *SFMBT1* encoding a drosophila scm-like protein is potentially involved in the transcriptional repression of homeotic genes in embryonic development via the histone methylation mechanism. Our study shows that it may play a novel role in hypertension, but its disease causing mechanism should be further elucidated [60]. Although no genes identified by us were significantly confirmed by the WTCCCHS data (Caucasian), *COMMD7*, which encodes COMM domain-containing protein 7 on 20q11.21, was replicated with a borderline significance (pFDR_LRC_ = 7.46×10^−2^). *COMMD* and *COMM* domains 1–10 are extensively conserved in multicellular eukaryotic organisms and define a novel family of structural and functional homologs of *MURR1/COMMD1* that suppress NF-kappaB by affecting the association of NF-kappaB with chromatin [61]. *MURR1/COMMD1* is also a regulator of epithelial sodium channels, and therefore may regulate blood pressure [62].

Some potential hypertension susceptibility genes were identified by our genome-wide GBAS analysis and gene expression analysis but were not replicated in the GBAS analysis of HKHS or WTCCCHS. For example, *TMEM56* on 1p21.3 and *KIAA1797* on 9p21 were simultaneously identified by all four regression models of our genome-wide GBAS. For these two genes, the smallest p values in the genome-wide GBAS of the four regression models were 6.78×10^−20^ and 1.03×10^−36^, respectively (**Table 2**), and the largest proportion that SNPs satisfy p<10^−2^ in a SBAS among the four regression models is 58.82% and 27.38%, respectively. These two genes were validated by our human gene expression analysis [*TMEM56*: p_ANCOVA_ = 5.77×10^−3^; *KIAA1797*: p_ANCOVA_ = 7.97×10^−3^] and *TMEM56* was also validated by the mice gene expression analysis [p_ANOVAaorta_ = 3.81×10^−3^; p_ANOVAheart_ = 4.48×10^−2^; p_ANOVAliver_ = 1.39×10^−2^] (**Table 2**). In addition, *FURIN* on 15q26.1, which encodes a subtilisin-like pro-protein convertase family protein [GBAS: p_CLRN_ = 3.62×10^−5^ (pFDR_CLRN_ = 2.04×10^−2^); p_CLRC_ = 3.34×10^−8^ (pFDR_CLRC_ = 1.52×10^−2^); p_CLRN,BMI_ = 4.76×10^−5^ (pFDR_CLRN,BMI_ = 2.16×10^−2^); p_CLRC,BMI_ = 8.66×10^−8^ (pFDR_CLRC,BMI_ = 1.52×10^−2^); gene expression: p_ANCOVA_ = 2.26×10^−3^] (**Table 2**), has been associated with blood pressure [63]. Due to the possibility of population specificity, these genes should be further studied even though they were not replicated in a GBAS of HKHS or WTCCCHS.

Our GBAS also identified some hypertension-associated genes that showed differential allelic distributions between hypertensive patients and normotensive controls and the genes could be replicated by the GBAS of HKHS, even though no significant differences in gene expression were observed. *ACTN4* on 19q13 is associated with cortical renal glomerulopathies, abnormal kidney physiology, small kidney, proteinuria, and an abnormal blood urea nitrogen level [64]. *CYP7B1* on 8q21.3 is associated with lipoproteins in humans [65] and is associated with abnormal cholesterol homeostasis in mice [66]. *FAS* on 10q24.1 is associated with hypertension [67], decreased body size, and increased body weight in mice [68], [69]. These SNPs may exert their effects via mechanisms other than by varying gene expression.

This study investigated the association between DNA-level variants (SNPs) and disease status of YOH by examining differential allelic distributions between patient and control groups and also investigated the association between mRNA-level variants (gene expression) and disease status of YOH. An interesting question to answer is whether SNP variants influence YOH development via gene expression regulation. Therefore, we examined whether the genes with differential allelic and expression distributions between patient and control groups in the present study were regulated by SNPs in the 2-Mb windows around the differentially expressed genes (i.e., cis-eQTL mapping). Under a less stringent criterion, 70 SNPs on 11 genes (*TMEM56*, *KIAA1797*, *LARS*, *GRB14*, *IGF1*, *WWOX*, *HLCS*, *CSF1*, *CSNK1G3*, *TTC12* and *SYNE2*) were identified to be cis-eQTL, generating a testable hypothesis that the 70 cis-eQTL may regulate hypertension-associated genes in turn to influence disease status.

Among the 11 genes, *GRB14*, which encodes a growth factor receptor binding protein on 2q22-q24, can interact with insulin receptors and insulin-like growth-factor receptors to modulate insulin signaling in mice [70], [71]. This gene was found to be associated using GBAS and gene expression analyses of the TWNHS, and had been identified in a previous GWAS of blood pressure in East Asian populations [28]. Moreover, some hypertension-associated cis-eQTL act as intronic enhancers at transcriptional factor binding sites on this gene. This protein likely has an inhibitory effect on receptor tyrosine kinase signaling and thus on the insulin receptor signaling pathway, through which *GRB14* variants may exert their effect on the development of hypertension. *IGF1*, another insulin-related gene which encodes insulin-like growth factor on 12q23.2, was associated with hypertension by GBAS and gene expression analyses of the TWNHS, successfully validated by GBAS of the HKHS in this study, and was previously associated with cardiovascular disorders [38], [39], [40], [41], [42] and metabolic disorders [43], [44], [45], [46], [47], [48], [49], [50], [51], [52]. Some hypertension-associated cis-eQTL act as intronic enhancers at transcriptional factor binding sites on this gene were also found. *WWOX* was found relevant by GBAS and gene expression analyses of the TWNHS, successfully validated by GBAS of the HKHS in this study, identified by a previous GWAS in hypertension in a Japanese population [34], and associated with hypertension-related traits in mice [59]. Moreover, some hypertension-associated cis-eQTL which act as intronic enhancers at transcriptional factor binding site on this gene were also found. *WWOX* encoded protein is more than 90 percent identical to the mouse protein responsible for modulation of tumor necrosis factor-alpha-induced apoptosis and therefore suggests an apoptosis correspondence for the human protein. This gene contains a short-chain dehydrogenase/reductase domain and is highly expressed in hormonally regulated normal tissues such as ovary, testis, and prostate. More recent evidence has suggested important roles of *WWOX* in the suppression of tumor growth, the regulation of a wide variety of cellular functions, steroid metabolism, and possibly hypertension [72], [73].

There were other cis-eQTL with relevant biological functions. For example, five cis-eQTL on *SYNE2* were found, where rs9944035 is a missense variant leading to non-conservative change, rs10140978 locates in promoter/regulatory region, and rs1890908 is a missense variant leading conservative change. Other examples include that rs10988 on *LARS* is a missense variant leading to conservative change and rs333970 on *LARS* is a splicing site. These cis-eQTL may play relevant roles in basic genetics of gene regulation to confer susceptibility to YOH by suppressing, inhibiting, enhancing or activating transcriptional levels of the identified YOH associated genes.

In addition to examine cis-eQTL for our identified hypertension-associated genes in **Table 2**, we also used the Genevar database [37] to investigate cis-eQTL for the hypertension-associated genes in **Table 1**. Results showed that the genes identified by our integrative analysis of SNP and gene expression did contain a higher proportion of hypertension-associated cis-eQTL than a SNP-only GWAS; the proportions of genes containing hypertension-associated cis-eQTL were 0.846 versus 0.273 and the proportions of hypertension-associated cis-eQTL SNPs in genes were 0.140 versus 0.019. This suggests the importance of an integrative analysis of SNP and gene expression in the post-GWAS era.

Our previous genome-wide association study of YOH analyzed 175 matched pairs of patients and normotensive controls genotyped with the Affymetrix Human Mapping 100 K Set in the Han Chinese population of Taiwan [29]. The study identified a significant genetic interaction between rs1886985 on chromosome 6 and rs6129969 on chromosome 20. In addition, the study also identified a significant SNP quartet composed of rs9308945, rs6711736, rs6729869 and rs10495809 on chromosome 2, using a sliding-window p-value combination method. We also attempted to evaluate the genetic association of the six SNPs in the current study of 400 matched pairs of YOH patients and normotensive controls genotyped with the Illumina HumanHap550-Duo BeadChip in the same Han Chinese population of Taiwan. SNPs rs6711736 and rs10495809 are on the Illumina HumanHap550-Duo BeadChip and SNPs rs1886985, rs6129969, rs9308945 and rs6729869 were imputed based on the 1,000 Genomes Project reference panel using IMPUTE2 [74]. Results show that genetic association between YOH and the SNP quartet composed of rs9308945, rs6711736, rs6729869 and rs10495809 on chromosome 2 was confirmed (p_CLRN_ = 1.79×10^−3^, p_CLRC_ = 1.31×10^−4^, and p_CLRBMI_ = 2.96×10^−5^), but genetic interaction between rs1886985 and rs6129969 was not significant in this TWNHS.

The original WTCCCHS study only performed SBAS and no significant SNPs or genes attained a genome-wide significance level of 5×10^−7^ [17]. In our WTCCCHS data analysis, we performed both SBAS and GBAS to examine the 100 significant genes identified by the genome-wide GBAS in our TWNHS. We made a comparison of SBAS and GBAS in our WTCCCHS data analysis based on the 87 genes (SNP data on 13 out of 100 significant genes were not available on the Affymetrix Human Mapping 500 K Set). At a false discovery rate of pFDR<0.05, GBAS identified three genes (*ACTN4*, *CYP7B1* and *PIP5K3*) and SBAS identified 2 genes (*ACTN4* and *TCF7L1*). The concordance rate of gene mapping results in SBAS and GBAS in our WTCCCHS data analysis is 96.55%.

GBAS has some merits compared with SBAS [35], [75], [76]. Because GBAS combines genetic contributions of multiple SNPs in a gene, the test power may be increased, and the effect of locus heterogeneity on gene mapping may be alleviated. In addition, the issue of over-correction with a large number of association tests in a genome-wide association study is mitigated because the number of association tests is reduced. Because the effect of the whole gene is evaluated, the association results can be directly interpreted using established gene functions and models. On the other hand, the gene is a natural unit in which a set of SNPs can be combined. Therefore, GBAS provides a convenient marker selection alternative to LD-based or sliding-window strategies for a genome-wide multilocus association scan. GBAS provides a convenient, feasible, and powerful tool for identifying genes involved in complex disorders.

Several limitations of this study should be noted when interpreting the results. First, the phenotype definition and sample ascertainment were not exactly the same in the three studies (TWNHS, HKHS, and WTCCCHS). For example, the YOH study in TWNHS recruited patients with hypertension with an age of onset ≤50, but this criterion was not considered in HKHS or WTCCCHS. Second, the three studies used different genome-wide oligonucleotide SNP arrays. Therefore, the SNP markers that were analyzed were different. Our study used the Illumina HumanHap550-Duo BeadChip; HKHS used the Illumina HumanHap610-quad BeadChip; WTCCCHS used the Affymetrix Human Mapping 500 K Set. Some association signals identified by TWNHS could not be examined in the other two studies due to a lack of SNPs in the regions of interest. Third, the controls in the WTCCCHS data analysis were the 3,004 shared community controls for seven complex diseases in WTCCC [17]. Some controls may not be normotensive and, therefore, the association signal may be diluted due to a misclassification of hypertensive patients to normotensive status. In other words, significant association identified by TWNHS may be not replicable in WTCCCHS due to the reduced power of WTCCCHS. Finally, our current gene expression analysis aimed to prioritize hypertension susceptibility genes for further confirmation. The sample size was somewhat small, although the selected samples had extremely discordant genotype distributions. The small sample size could lead to false negatives. Therefore, we used a liberal multiple-testing correction in the expression data analysis. In our next gene expression study, we will conduct a large-scale gene expression study of 400 patients with hypertension and 400 normotensive controls using the Human OneArray (Phalanx Biotech, Hsin-Chu, Taiwan), which provides ∼300,000 genome probes to achieve more power.

## Materials and Methods

### Ethics statement

Written informed consent was obtained from each participant at his/her initial clinic visit. The study was approved by the Internal Review Board of Academia Sinica (Permit Number: AS-IRB01-08012).

### DNA samples

This TWNHS analyzed 400 matched pairs of patients with hypertension and normotensive controls. Patients were recruited from the Academia Sinica Multi-Center YOH Genetic Study. A one-to-one match strategy for age (±5 years) and gender was applied to select 400 normal controls from the Taiwan Han Chinese Cell and Genome Bank [77]. Immortalized cell lines from their lymphocytes have been established. The criteria for sample inclusion/exclusion and sample matching are described in our previous GWAS [29]. Genetic association replication analysis was performed using data from the other Han Chinese population in Hong Kong (HKHS) [36] and a Caucasian population (WTCCCHS) [17]. The HKHS collected 111 families with a total of 315 individuals, including 37 affected sib pairs and 195 discordant sib pairs. SBP, DBP, MBP off treatment, and disease status of hypertension were measured. The WTCCCHS collected 1,999 patients with hypertension and 3,004 normal controls consisting of 1,504 samples from the 1958 British Birth Cohort and 1,500 samples from the UK Blood Service Control Group [17].

### SNP genotyping experiments

The genomic DNA of 800 Han Chinese samples was isolated from leukocytes using a Puregene kit (Gentra Systems, Minneapolis, MN, USA) for genomic DNA isolation. The DNA concentration was quantified and adjusted to 60 ng/µl using a NanoDrop ND-1000 Spectrophotometer (NanoDrop Technologies, DE, USA). All samples were genotyped by deCODE Genetics (Reykjavik, Iceland) with the Illumina HumanHap550-Duo BeadChip (Illumina, Inc., San Diego, CA, USA). This SNP genotyping platform contains 560,186 tagging SNPs selected from phases I and II of the HapMap Project [78], [79], [80], [81]. Genotype calling was performed using the Illumina BeadStudio Genotyping Module (Illumina, Inc.). Samples in the HKHS were genotyped with the Illumina HumanHap610-quad BeadChip (Illumina, Inc.), which enables the whole-genome genotyping of 620,901 SNPs. Samples in the WTCCCHS were genotyped with the Affymetrix Human Mapping 500 K Set (Affymetrix, Inc., San Diego, CA, USA), which provides 500,568 SNPs. The genotyping experiment was described previously [17].

### Gene expression microarray experiments

A total of 12 matched pairs of patients with hypertension with the highest risk scores and normotensive controls with the lowest risk scores were selected. Total RNA was extracted using TRIzol Reagent (Life Technologies, Inc., CA, USA) and then reverse transcribed to cDNA and cRNA using Illumina TotalPrep™ RNA Amplification Kit (Illumina, Inc.). The cRNA concentration was quantified and adjusted to 150 ng/µl using a NanoDrop ND-1000 Spectrophotometer (NanoDrop Technologies, DE, USA). Gene expression experiments were performed by Genetech Biotech (Taipei, Taiwan) with the Illumina HT12 Expression BeadChip (Illumina, Inc.). This gene expression platform contains more than 25,000 annotated genes and more than 48,000 probes derived from the NCBI RefSeq (Build 36.2, Rel 22) and the UniGene (Build 199) databases. We also analyzed the gene expression data sets of genetically hypertensive and normotensive inbred mouse strains. Tissues of aorta, heart, kidney and liver from the two mouse strains were collected. The details about tissue collection, RNA preparation, and microarray experiment was described previously [82].

### Statistical analyses

A flowchart for the overall study design is provided (**Figure 1**). Quality of samples and SNPs were examined and population stratification in the TWNHS was evaluated. Genome-wide SBAS and GBAS were performed and confirmed by microarray gene expression analyses for the Han Chinese population of Taiwan. The identified genes were further examined by two association studies, the HKHS and the WTCCCHS. The detailed analysis procedures are as follows.

First, sample and SNP quality were examined. We checked the consistency of self-reported gender and X chromosome, calculated the GCR, estimated MAF using an allele counting approach, and examined HWE using an exact HWE test [83] with SAS GENETICS (SAS Institute, Inc., Cary, NC, USA) for all 546,376 autosomal SNPs from 560,184 tag SNPs probed on the HumanHap550-Duo BeadChip. Second, population stratification and admixture were examined. We evaluated population structure using a genomic control method [84] with SAS GENETICS (SAS Institute, Inc.) and a principal component method with EigenStrat software [85]. A non-parametric median test was used to examine differences of principal components in case and control groups [86].

Third, genome-wide SBAS was performed based on conditional logistic regression analyses using SAS STAT (SAS Institute, Inc.). The analysis regressed a dichotomous response on a nominal SNP coding for a genotype-based analysis or a continuous SNP coding for a trend-based analysis at each SNP locus with or without a covariate adjustment for BMI, resulting in four models: (1) conditional logistic regression with a nominal genotype without BMI adjustment (“CLR_N_”), (2) conditional logistic regression with a continuous genotype without BMI adjustment (“CLR_C_”), (3) conditional logistic regression with a nominal genotype and BMI adjustment (“CLR_N,BMI_”), and (4) conditional logistic regression with a continuous genotype and BMI adjustment (“CLR_C,BMI_”). For each SNP locus, p-values of a one degree-of-freedom Wald test were calculated for model CLR_C_ and CLR_C,BMI_, and p-values of a two degree-of-freedom Wald test were calculated for model CLR_N_ and CLR_N,BMI_.

Fourth, genome-wide GBAS was performed. According to SNP- and gene-annotation provided by the SNPper database [87], all 475,157 validated SNPs were divided into 216,994 intragenic SNPs on 15,373 genes and 258,163 intergenic SNPs. If a SNP mapped to more than one gene, it was assigned randomly. The mean, median, and standard deviation of the number of SNPs within a gene were 14.12, 6.00, and 32.19, respectively. In summary, 1,688 genes (10.98%), 9,000 genes (58.57%), 4,387 genes (28.55%), 291 genes (1.89%), and 1 gene (<0.01%) contained 1, 2–10, 11–100, 101–1000, and >1000 SNPs; *CSMD1* on chromosome 8 contained the most SNPs (1,518) of any gene.

In the first step (prioritization test) of GBAS, we prioritized genes using a more stringent criterion (pFDR<10^−3^) to reduce the potential inflated significance. For each of the 15,373 genes, with a specific regression model, p-values of intragenic SNPs within the same gene were combined by calculating a truncated product p-value test statistic [88], [89], [90] with a p-value truncation threshold of 0.05 to represent a gene [35]. An FDR [91] was calculated using SAS GENETICS (SAS Institute, Inc.) to account for a multiple test correction. Genome-wide statistical significance of gene-based association tests was evaluated by adjusting for multiple tests of all genes using a stringent threshold, pFDR<10^−3^. The identified candidate genes were further validated in the next step by using a permutation test for a stringent consideration. In the second step (permutation test) of GBAS, we used the threshold conventionally adopted (pFDR<5×10^−2^) to further remove the inflated significance in the first test and take p-value dependency into consideration. Then, 10,000 permutations that we permuted for disease status but that maintained the matched relationship between patients and controls were performed for calculation of empirical p-values. In each permutation sample, we determined the statistical significance of the identified genes using the same gene-based association test. Empirical p-values of each identified gene were calculated and adjusted by FDR, where FDR was calculated across four models (“CLR_N_”, “CLR_C_”, “CLR_N,BMI_” and “CLR_C,BMI_”) jointly. For association validation of the identified genes, statistical significance was claimed under a significance threshold of pFDR<5×10^−2^. The details of our genome-wide GBAS method are described in **Method S1**.

Fifth, microarray gene expression analysis was performed. Based on the genes identified in the second step, we selected 12 patients and 12 controls with extremely discordant genotypic distributions between the patient and control groups and conducted a microarray gene expression study using the Illumina HT-12 Expression Beadchip (Illumina, Inc.). Genotypes of all SNPs in the genes identified in the second step were assigned as “risky genotypes” or “protective genotypes” if the genotype frequencies in case group were higher or lower than in control group, respectively. The average proportion of risky genotypes in every patient and average proportion of protective genotypes in every control were calculated for every control over the identified genes. The 12 patients who carried the maximum proportion of risky genotypes and the 12 controls who carried the maximum proportion of protective genotypes among all samples were selected. The details of the selection of extremely discordant case-control pairs are described in **Method S2**. Quantile normalization [92] was applied to normalize gene expression, and analysis of covariance (ANCOVA) with an adjustment for BMI using PARTEK software (Partek, Inc., St. Louis, Missouri, USA) was performed to analyze the normalized data and identify the differentially expressed genes among the 100 identified genes. Confirmation of genetic association was claimed if p_ANCOVA_<5×10^−2^. The identified differentially expressed genes were used to perform a cluster analysis with an average linkage in which distances of any two samples were measured by Kendall's dissimilarity [93]. Except for two genes, *KIAA1797* and *AP3S1*, not available in the mouse gene expression study [82], analysis of variance (ANOVA) using SAS STAT (SAS Institute, Inc.) was performed to discuss tissue-specific variation in gene expression between genetically hypertensive and normotensive mouse strains. Confirmation of genetic association was claimed if p_ANOVA_<5×10^−2^.

Sixth, we performed gene-centric cis-acting expression quantitative trait loci (cis-eQTL) analysis by using the lymphoblastoid cell line datasets in Geneva GenCord [94] and MuTHER [95] provided in the Genevar (GENe Expression VARiation) [37]. The analysis examined the association between gene expressions and SNPs in 2-Mb windows around the differentially expressed genes identified in our gene expression study (**Table 2**) and hypertension-associated genes identified in previous hypertension genomic studies (**Table 1**); Spearman's correlation coefficient and correlation test of gene expressions and continuous SNP coding were calculated. Cis-eQTL with p<5×10^−2^ were selected. Furthermore, we examined if the cis-eQTL were also associated with YOH in the TWNHS; conditional logistic regression at each cis-eQTL was performed with or without a covariate adjustment for BMI by using SAS STAT (SAS Institute, Inc.), where a continuous SNP coding was considered. YOH-associated Cis-eQTL with p<5×10^−2^ were selected. Finally, we examined the direction of genetic effect (risky effect or protective effect) and gene regulation (up-regulation or down-regulation) of the disease associated alleles of cis-eQTL.

Finally, we conducted GBAS for genomic data sets of a Han Chinese population of Hong Kong and a Caucasian population. We analyzed genotype data of SNPs on the 100 identified genes and four phenotypes from the HKHS GWAS. The four phenotypes were: (1) dichotomous disease status, (2) quantitative SBP, (3) quantitative DBP, and (4) quantitative MBP. A single-locus sib-based transmission disequilibrium test (sib-TDT) [96] was applied to examine the genetic association of the dichotomous disease status of hypertension with the SNPs. A quantitative family-based association test (QFAM) [97] was applied to examine the genetic association of quantitative SBP, DBP, MBP, and genes (“QFAM_SBP_”, “QFAM_DBP_”, and “QFAM_MBP_”) by calculating p-values for a within-family component. All the analyses were done with PLINK software [97]. Moreover, we permuted the disease status to obtain 10,000 permutation samples for calculation of empirical p-values. In each permutation sample, we examined the statistical significance of the identified genes using the same gene-based association test described in the fourth step. The empirical p-value of each identified gene was calculated and adjusted by FDR, where FDR was calculated across four phenotypic models (“sib-TDT”, “QFAM_SBP_”, “QFAM_DBP_”, and “QFAM_MBP_”) jointly.

Replication of genetic association was claimed if pFDR<5×10^−2^. We also analyzed genotype data from the WTCCCHS [17]. Single-locus association tests were performed using logistic regression analyses, which regressed a dichotomous response on continuous or nominal-coding genotypes at each SNP locus with or without covariate adjustment for gender, resulting in four logistic regression models: (1) logistic regression with a nominal genotype without gender adjustment (“LR_N_”), (2) logistic regression with a continuous genotype without gender adjustment (“LR_C_”), (3) logistic regression with a nominal genotype with gender adjustment (“LR_N,Gender_”), and (4) logistic regression with a continuous genotype with gender adjustment (“LR_C,Gender_”). Again, we permuted the disease status to obtain 10,000 permutation samples for calculation of empirical p-values. In each permutation sample, we determined the statistical significance of the identified genes using the same gene-based association test described in the fourth step. The empirical p-value of each identified gene was calculated and adjusted by FDR, where FDR was calculated across four models (“LR_N_”, “LR_C_”, “LR_N,Gender_”, and “LR_C,Gender_”) jointly. Replication of genetic association was claimed if pFDR<5×10^−2^. Note that 13 genes out of 100 were not examined in the WTCCCHS because the Affymetrix Human Mapping 500 K Set does not provide SNPs on these 13 genes.

### Bioinformatics analyses

We used the Genetic Association Database (GAD, http://geneticassociationdb.nih.gov/cgi-bin/index.cgi) and Online Mendelian Inheritance in Man (OMIM, http://www.ncbi.nlm.nih.gov/omim/) to determine whether the identified genes with differential allelic associations and differential expression in patients with hypertension and normotensive controls were also reported. In addition, we used Mouse Genome Informatics (MGI, http://www.informatics.jax.org/) to determine if the identified genes were involved in hypertension-related phenotypes in mice and other mammals.

## Supporting Information

Figure S1 **Principal component analysis for evaluation of the population substructure in patient and control groups.** Hypertensive patients (symbol: ○) and normotensive controls (symbol: ×) are projected onto a two-dimensional plane defined by the first two eigenvectors.
(DOC)

Figure S2 **P-values (−log_10_ scale) of genome-wide SBAS.** (A) P-values of the conditional logistic regression with a nominal genotype without BMI adjustment (CLR_N_). (B) P-values of the conditional logistic regression with a nominal genotype with BMI adjustment (CLR_N,BMI_). (C) P-values of the conditional logistic regression with a continuous genotype without BMI adjustment (CLR_C_). (D) P-values of the conditional logistic regression with a continuous genotype with BMI adjustment (CLR_C,BMI_). In each figure, the vertical axis is the raw p-values (−log_10_ scale) of gene-based association tests, and the horizontal axis is cumulative physical position (Mb scale).
(DOC)

Table S1 **Raw p-values (−log_10_ scale) of 101 genes identified by the genome-wide GBAS.** Gene name, number of SNPs, chromosome, physical position, and category (the 101 genes are classified into 12 categories (Category I to XII) according to gene significance in the four genome-wide GBAS models) are shown. The raw p-values in −log10 scale are shown for our GBAS of the TWNHS (Method: CLR_N_, CLR_C_, CLR_N,BMI_, and CLR_C,BMI_) and gene expression analysis (Method: ANCOVA) in order. Raw p-values that reached pFDR<5×10^−2^ in each model are marked in bold.
(XLS)

Table S2 **The information for the 101 identified hypertension susceptibility genes collected from the GAD, OMIM, and MGI databases.**
(XLS)

Table S3 **Beta coefficients and their standard errors for the 17 differentially expressed genes in hypertension.**
(DOC)

Table S4 **The cis-eQTL analysis of 17 genes in** **Table 2** **identified in the present study.** Gene name followed by chromosome number, RS number of cis-eQTL, beta coefficient in CLR_C_, beta coefficient in CLR_C,BMI_, p-values of the tests of beta coefficients, the maximal Spearman correlation coefficient from Geneva GenCord and MuTHER, p-value of the test of Spearman correlation, and the first and the second allele followed by an index for the direction of genetic effect and gene regulation of the YOH-associated risky alleles in the cis-eQTL (r/u, r/d, p/u and p/d denote that risky/up-regulated, risky/down-regulated, protective/up-regulated and protective/down-regulated, respectively). “NS” indicates that beta coefficient is not significant and p-value is not shown. “NC” indicates that the positive/negative of Spearman correlation coefficients from Geneva GenCord and MuTHER is not consistent.
(XLS)

Table S5 **The cis-eQTL analysis of 54 genes in** **Table 1** **identified by previous GWAS in hypertension.** Gene name followed by chromosome number, RS number of cis-eQTL, beta coefficient in CLR_C_, beta coefficient in CLR_C,BMI_, p-values of the tests of beta coefficients, the maximal Spearman correlation coefficient from Geneva GenCord and MuTHER, p-value of the test of Spearman correlation, and the first and the second allele followed by an index for the direction of genetic effect and gene regulation of the YOH-associated risky alleles in the cis-eQTL (r/u, r/d, p/u and p/d denote that risky/up-regulated, risky/down-regulated, protective/up-regulated and protective/down-regulated, respectively). “NS” indicates that beta coefficient is not significant and p-value is not shown. “NC” indicates that the positive/negative of Spearman correlation coefficients from Geneva GenCord and MuTHER is not consistent.
(XLS)

Method S1 **Genome-wide gene-based association tests.**
(DOC)

Method S2 **Selection of extremely discordant case-control pairs.**
(DOC)
