# Supplementary material for: Identification of IGF1, SLC4A4, WWOX, and SFMBT1 as Hypertension Susceptibility Genes in Han Chinese with a Genome-Wide Gene-Based Association Study
Source: PLoS One. 2012 Mar 29;7(3):e32907. doi: 10.1371/journal.pone.0032907 (PMC3315540; doi:10.1371/journal.pone.0032907)

**Figure S1. Principal component analysis for evaluation of the population substructure in patient and control groups.** Hypertensive patients (symbol: ○) and normotensive controls (symbol: ×) are projected onto a two-dimensional plane defined by the first two eigenvectors.


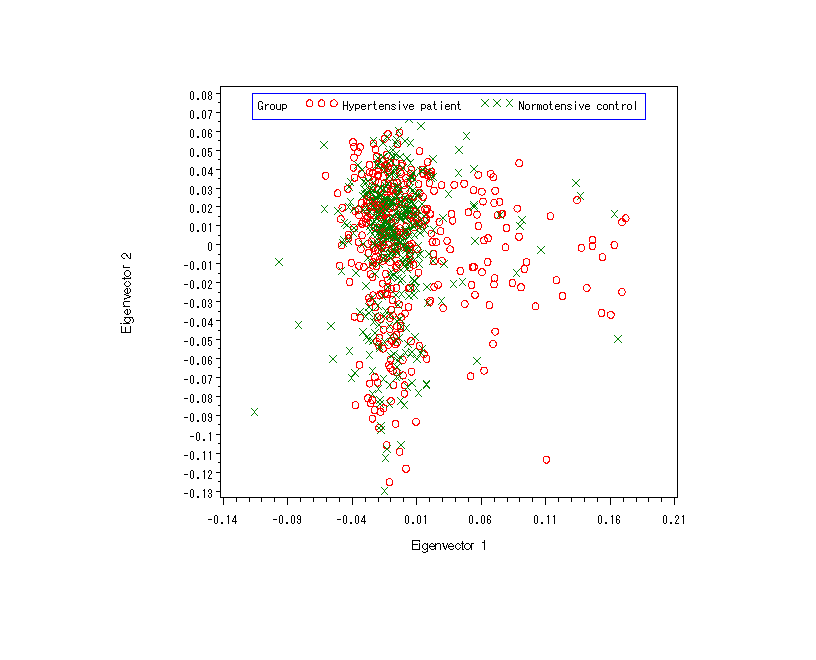

Supplement: Figure S1 — Principal component analysis for evaluation of the population substructure in patient and control groups. Hypertensive patients (symbol: ○) and normotensive controls (symbol: ×) are projected onto a two-dimensional plane defined by the first two eigenvectors. (DOC) [file pone.0032907.s001.doc]
