# Supplementary material for: Identification of IGF1, SLC4A4, WWOX, and SFMBT1 as Hypertension Susceptibility Genes in Han Chinese with a Genome-Wide Gene-Based Association Study
Source: PLoS One. 2012 Mar 29;7(3):e32907. doi: 10.1371/journal.pone.0032907 (PMC3315540; doi:10.1371/journal.pone.0032907)

**Figure S2.** **P-values (–log_10_ scale) of genome-wide SBAS.** (A) P-values of the conditional logistic regression with a nominal genotype without BMI adjustment (CLR_N_). (B) P-values of the conditional logistic regression with a nominal genotype with BMI adjustment (CLR_N,BMI_). (C) P-values of the conditional logistic regression with a continuous genotype without BMI adjustment (CLR_C_). (D) P-values of the conditional logistic regression with a continuous genotype with BMI adjustment (CLR_C,BMI_). In each figure, the vertical axis is the raw p-values (–log_10_ scale) of gene-based association tests, and the horizontal axis is cumulative physical position (Mb scale).

(A)


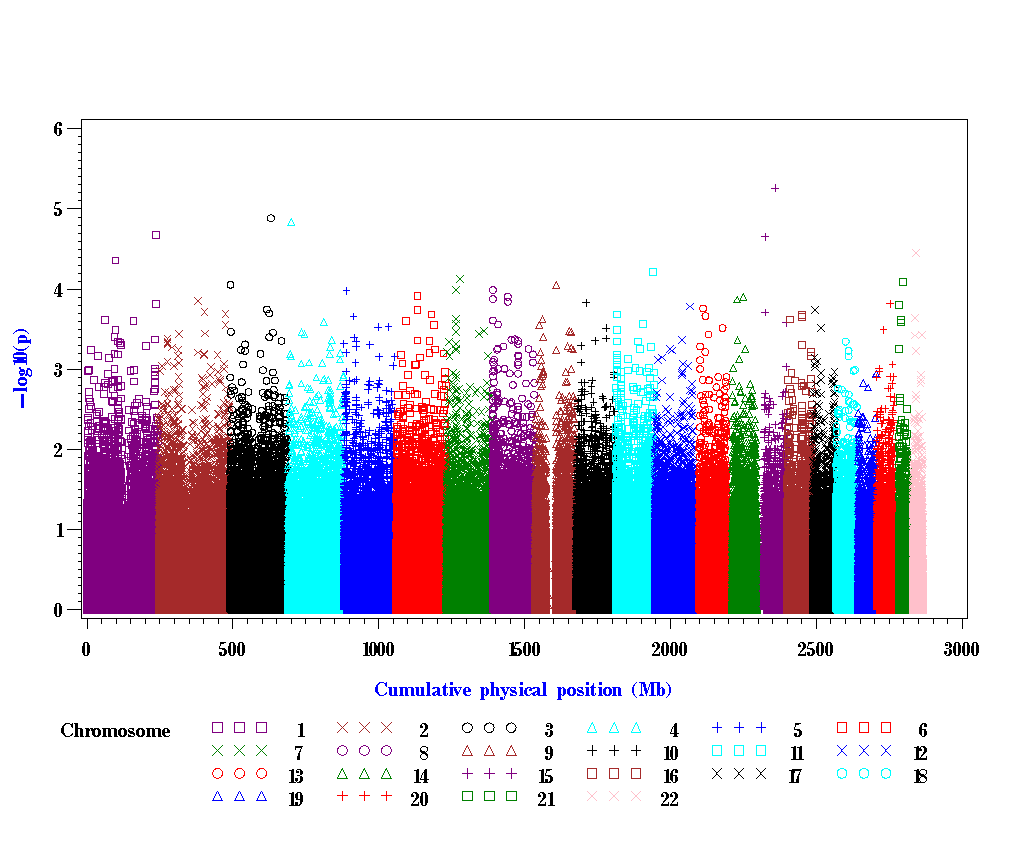


(B)


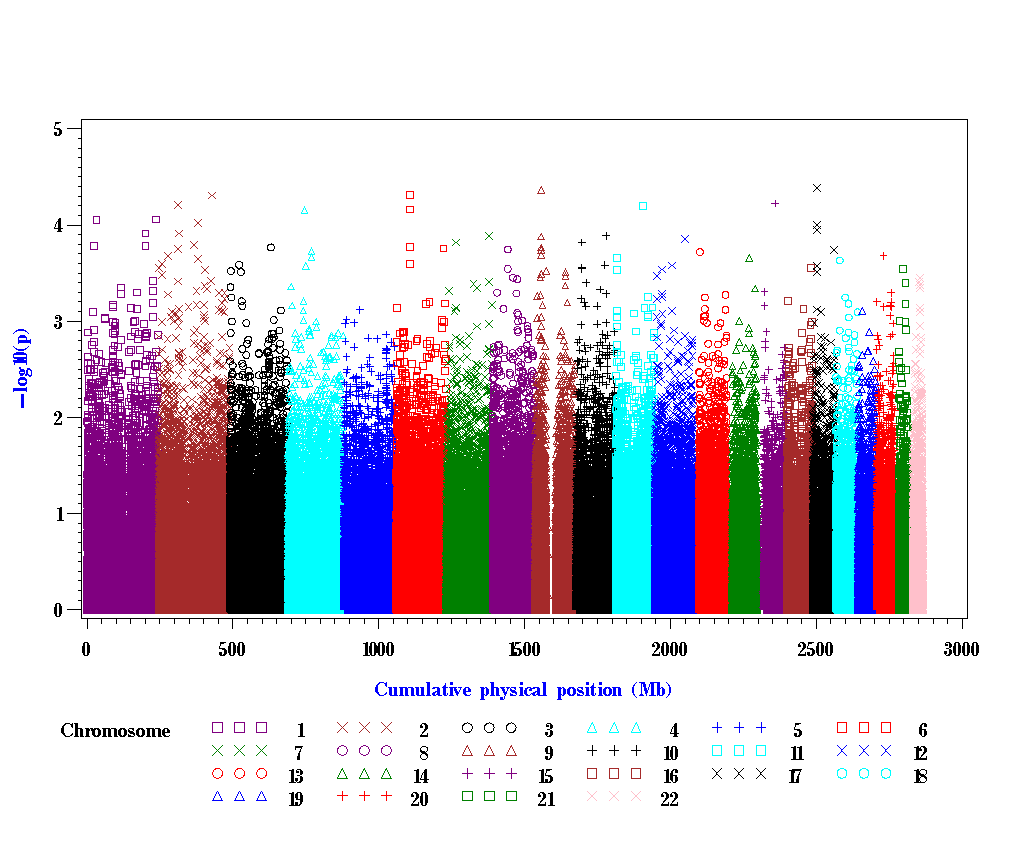


(C)


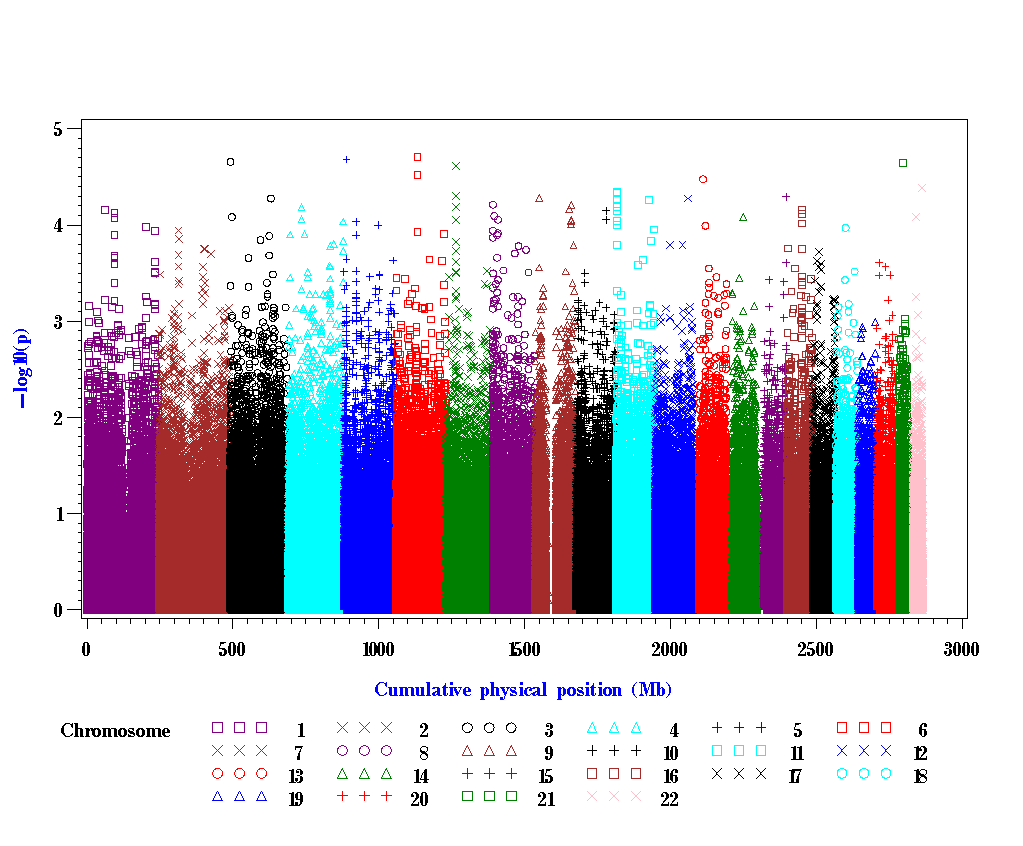


(D)


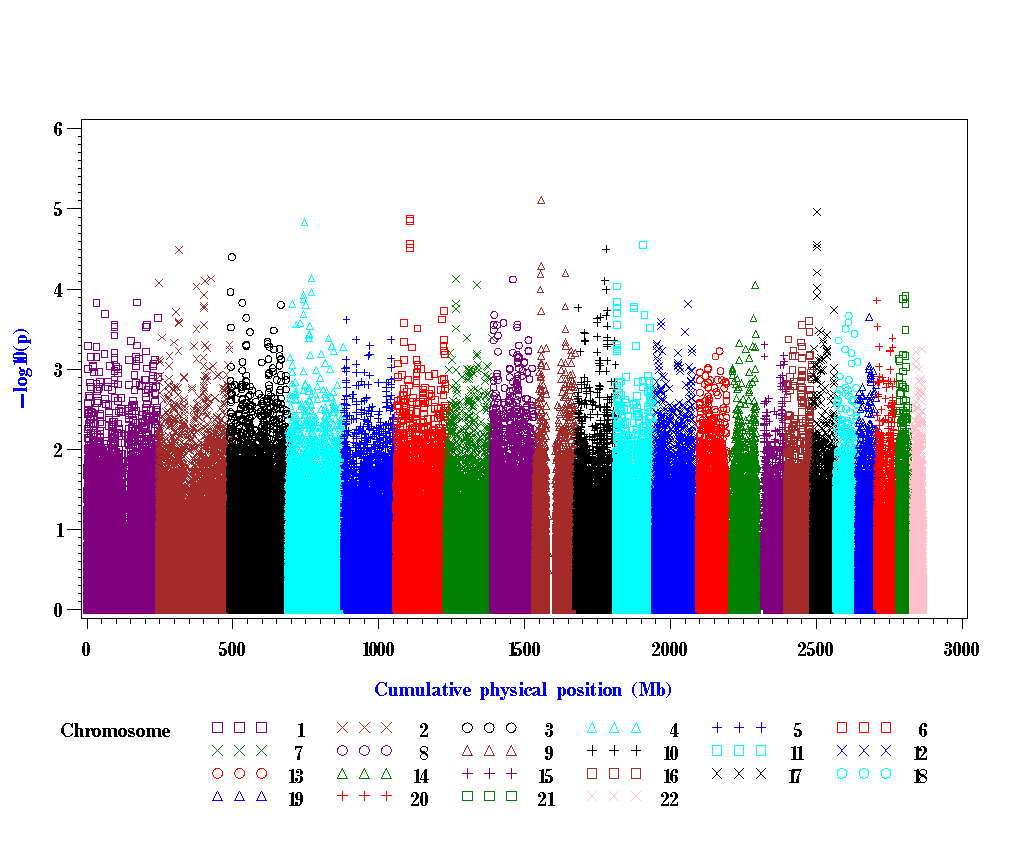

Supplement: Figure S2 — P-values (−log10 scale) of genome-wide SBAS. (A) P-values of the conditional logistic regression with a nominal genotype without BMI adjustment (CLRN). (B) P-values of the conditional logistic regression with a nominal genotype with BMI adjustment (CLRN,BMI). (C) P-values of the conditional logistic regression with a continuous genotype without BMI adjustment (CLRC). (D) P-values of the conditional logistic regression with a continuous genotype with BMI adjustment (CLRC,BMI). In each figure, the vertical axis is the raw p-values (−log10 scale) of gene-based association tests, and the horizontal axis is cumulative physical position (Mb scale). (DOC) [file pone.0032907.s002.doc]
