# Supplementary material for: Identification of IGF1, SLC4A4, WWOX, and SFMBT1 as Hypertension Susceptibility Genes in Han Chinese with a Genome-Wide Gene-Based Association Study
Source: PLoS One. 2012 Mar 29;7(3):e32907. doi: 10.1371/journal.pone.0032907 (PMC3315540; doi:10.1371/journal.pone.0032907)
Supplement: Table S3 — Beta coefficients and their standard errors for the 17 differentially expressed genes in hypertension. (DOC) [file pone.0032907.s005.doc]

**Table S3.** **Beta coefficients and their standard errors for the 17 differentially expressed genes in hypertension.**

| Gene information ^a^ | | | | Beta coefficient (s.e) of the human and mouse GE analysis ^b, c^ | | | | |
| --- | --- | --- | --- | --- | --- | --- | --- | --- |
| Gene | #SNPs | Chr | PP | Human | Mouse aorta | Mouse heart | Mouse kidney | Mouse liver |
| *TMEM56* | 17 | 1 | 95359361 | **0.193 (0.055)** | **42.516 (11.887)** | **-0.724 (0.305)** | -36.284 (16.001) | **144.691 (46.141)** |
| *KIAA1797* | 84 | 9 | 20638805 | **0.379 (0.115)** | NA ^d^ | NA ^d^ | NA ^d^ | NA ^d^ |
| *SFMBT1* | 23 | 3 | 52923372 | **-0.200 (0.089)** | **107.070 (33.384)** | 1.383 (3.692) | 7.835 (4.22) | -2.335 (5.105) |
| *LARS* | 6 | 5 | 145480189 | **0.362 (0.107)** | -24.880 (131.953) | 47.131 (23.301) | -5.809 (10.729) | -8.461 (5.131) |
| *GRB14* | 23 | 2 | 165054838 | **0.38 (0.113)** | 33.394 (48.423) | **-64.465 (25.962)** | **16.767 (6.648)** | 28.275 (21.299) |
| *IGF1* | 18 | 12 | 101305009 | **-0.221 (0.057)** | **-90.187 (37.42)** | -3.121 (1.405) | -3.735 (2.07) | **-**175.913 (116.84) |
| *FURIN* | 5 | 15 | 89205709 | **0.421 (0.103)** | -64.043 (91.244) | 14.809 (9.868) | 8.316 (15.461) | -3.926 (8.346) |
| *WWOX* | 422 | 16 | 76681421 | **-0.248 (0.071)** | **35.039 (13.31)** | -8.496 (4.56) | -6.29 (4.916) | **-16.344 (4.134)** |
| *HLCS* | 54 | 21 | 37056136 | **0.227 (0.076)** | **6.859 (2.982)** | **9.525 (2.987)** | 4.981 (3.272) | **15.177 (3.343)** |
| *CSF1* | 11 | 1 | 110251700 | **0.175 (0.068)** | **173.746 (52.785)** | -10.196 (32.249) | **-12.433 (4.052)** | -4.746 (4.476) |
| *CSNK1G3* | 17 | 5 | 122878220 | **0.403 (0.102)** | **-249.480 (58.52)** | **0.901 (0.235)** | **-68.102 (8.025)** | -8.187 (4.407) |
| *COMMD7* | 5 | 20 | 30776577 | **0.242 (0.093)** | **81.215 (36.267)** | 0.222 (0.477) | 0.858 (1.312) | -4.816 (18.264) |
| *AP3S1* | 8 | 5 | 115216213 | **-0.389 (0.118)** | NA ^d^ | NA ^d^ | NA ^d^ | NA ^d^ |
| *SLC4A4* | 33 | 4 | 72439536 | **0.095 (0.038)** | **88.728 (17.894)** | **-14.243 (3.055)** | **-256.621 (107.612)** | 9.858 (8.297) |
| *LACE1* | 15 | 6 | 108717334 | **0.147 (0.052)** | **232.918 (65.958)** | -12.448 (23.929) | 12.804 (11.462) | -1.333 (2.309) |
| *TTC12* | 18 | 11 | 112685412 | **0.185 (0.069)** | **101.390 (45.784)** | **49.900 (3.506)** | 1.259 (1.103) | -0.258 (0.473) |
| *SYNE2* | 65 | 14 | 63385073 | **0.123 (0.042)** | 18.766 (12.367) | 1.231 (1.626) | **125.736 (45.627)** | **8.012 (2.821)** |

^a^ Gene information includes gene name, the number of intra-genic SNPs [#SNPs], chromosome [Chr], and physical position [PP].

^b^ P-values of genes that reached p < 0.05 in gene expression studies are marked in bold.

^c^ A positive (negative) beta coefficient signifies that the gene expression in case group is higher (lower) than in control group.

^d^ NA denotes that expression data of a gene were not available in the mouse gene expression analysis.
