# Supplementary material for: Identification of IGF1, SLC4A4, WWOX, and SFMBT1 as Hypertension Susceptibility Genes in Han Chinese with a Genome-Wide Gene-Based Association Study
Source: PLoS One. 2012 Mar 29;7(3):e32907. doi: 10.1371/journal.pone.0032907 (PMC3315540; doi:10.1371/journal.pone.0032907)
Supplement: Method S1 — Genome-wide gene-based association tests. (DOC) [file pone.0032907.s008.doc]

# Method S1. Genome-wide gene-based association tests

# We illustrate the flow of the applied two-stage genome-wide gene-based association test as follows. At the first stage, we calculate p-values of single-locus association tests for all autosomal SNPs based on conditional logistic regression analyses using SAS STAT (SAS Institute, Inc.). The second stage consists of prioritization test and permutation test. We carry out a genome-wide gene-based association scan by using our algorithm as follows:

# (1) Prioritization test - all intra-gene SNPs are collected according to SNP- and gene-annotation of the Illumina HumanHap550-Duo BeadChip. Intra-gene SNPs within the same gene are collected as a SNP set, and the p-value of the *i*th intra-gene SNP within the *j*th gene are denoted as $\{p_{i,j},i=1,\cdots,i_{j},j=1,\cdots,J\}$, where $i_{j}$denotes the number of SNPs within the *j*th gene and *J* denotes the total number of genes studied. We combine p-values of intra-gene SNPs within a gene by using the truncated product p-value test statistic [1 – 4] expressed as follows:

$Z_{j}=\prod_{1\leq i\leq i_{j}} (p_{i,j})^{I[p_{i,j}<\lambda]},j=1,\cdots,J$,

where $\lambda$ is a pre-specified threshold of p-value truncation ($\lambda$=0.05 was used in this study) and *I*[·] denotes an indicator function. Under the assumption that all p-values are independent, p-values of genes $\{p_{j}^{\text{*}},j=1,\cdots,J\}$are calculated by using the exact cumulative distribution function (see Equation (2) in Reference [1]). To account for a multiple test correction, a false discovery rate (FDR) procedure [4] is used to calculate FDR-adjusted p-value, $\{pFDR_{j}^{*},j=1,\cdots,J\}$, using SAS GENETICS (SAS Institute, Inc.). Significant genes are identified using a threshold of$pFDR<0.001$. Suppose that we identify *K* candidate genes at the stage of gene prioritization.

(2) Permutation test - because the aforementioned analysis is performed under the assumption of “all p-values are independent”, the genes may be identified due to false positive. Therefore, a permutation test is employed to verify the significance of the *K* identified candidate genes. In total, 10,000 permutations are performed for calculation of empirical p-values. In the th permutation sample, we determine the statistical significance of the *K* identified genes using the same gene-based association test, . Empirical p-values of each identified gene are calculated as follows:

.

Empirical p-values are adjusted by an FDR procedure over the *K* previously identified genes [4] to obtain FDR-adjusted p-values, $\{pFDR_{j}^{**},j=1,\cdots,K\}$. For association confirmation of the previously identified genes, statistical significance is concluded using a significance threshold of$pFDR^{**}<0.01$.

# References

1. Zaykin DV, Zhivotovsky LA, Westfall PH and Weir BS: Truncated product method for combing p-values. *Genet Epidemiol* 2002, 22: 170-185.
2. Yang HC, Lin CY and Fann CSJ: A sliding-window weighted linkage disequilibrium test. *Genet Epidemiol* 2006, 30: 531-545.
3. Yang HC, Hsieh HY and Fann CSJ: Kernel-based association test. *Genetics* 2008, 179: 1057-1068.
4. Yang, HC, Liang, YJ, Chung, CM, Chen, JW and Pan, WH (2009/12). Genome-wide gene-based association study. *BMC Proceedings* 2009, 3: S135.
5. Benjamini Y and Hochberg Y: Controlling the false discovery rate: A practical and powerful approach to multiple testing. *JRSS – B* 1995, 57: 289-300.
