# Supplementary material for: Identification of IGF1, SLC4A4, WWOX, and SFMBT1 as Hypertension Susceptibility Genes in Han Chinese with a Genome-Wide Gene-Based Association Study
Source: PLoS One. 2012 Mar 29;7(3):e32907. doi: 10.1371/journal.pone.0032907 (PMC3315540; doi:10.1371/journal.pone.0032907)

# Method S2. Selection of extremely discordant case-control pairs

# We illustrate our selection procedures of case-control pairs which have an extremely discordant genotypic distribution between groups. Let *N_d_* and *N_c_* denote the total number of samples in disease and control groups in the study, respectively, and *N* = *N_d_* + *N_c_* (e.g., *N_d_* = 400 and *N_c_* = 400 in this study). Let *G* denote the number of identified genes (e.g., our GBAS identified 100 genes), and *S_g_* is the number of SNPs in the *g*th gene. For all SNPs in the identified genes, genotypes are assigned as “risky genotypes” if their genotype frequencies in disease group are higher than in control group. Genotypes are assigned as “protective genotypes” if the genotype frequencies in control group are higher than in disease group. For the disease group, let $X=\{X_{i,g,s},i=1,\cdots,N_{d},g=1,\cdots,G,s=1,\cdots,S_{g}\}$, where *X_i_*,*_g_*,*_s_* = 1 if the *i*th patient carries a risky genotype for the *s*th SNP in the *g*th gene; otherwise, *X_i_*,*_g_*,*_s_* = 0. Similarly, for the control group, let $Y=\{Y_{j,g,s},j=1,\cdots,N_{c},g=1,\cdots,G,s=1,\cdots,S_{g}\}$, where *Y_j_*,*_g_*,*_s_* = 1 if the *j*th normal control carries a protective genotype for the *s*th SNP in the *g*th gene; otherwise, *Y_j_*,*_g_*,*_s_* = 0. Based on the data of *X* and *Y*, three methods are used to select extremely discordant case-control pairs.

- **The first method:** We calculate the average proportion of risky SNPs over the identified genes for each patient as follows:

$p_{i}=\sum_{g=1}^{G} p_{i,g}/G,i=1,\cdots,N_{d}$,

where $p_{i,g}=\sum_{s=1}^{S_{g}} X_{i,g,s}/S_{g}$denotes the proportion of risky SNPs in the *g*th gene. Similarly, the average proportion of protective SNPs over the identified genes for each normal control can be written as follows:

$q_{j}=\sum_{g=1}^{G} q_{j,g}/G,j=1,\cdots,N_{c}$,

where $q_{j,g}=\sum_{s=1}^{S_{g}} Y_{j,g,s}/S_{g}$denotes the proportion of protective SNPs in the *g*th gene. Let $\tilde{p}_{i}=rank\{p_{1},\cdots,p_{N_{d}}\}$and $\tilde{q}_{j}=rank\{q_{1},\cdots,q_{N_{c}}\}$, where high values of $p_{i}$ and $q_{j}$will be assigned as high rank values. Then we identify cases and normal controls with the high rank values, respectively.

- **The second method:** We calculate the average rank of the proportion of risky SNPs for each patient as follows:

$s_{i}=\sum_{g=1}^{G} s_{i,g}/G,i=1,\cdots,N_{d}$,

where $s_{i,g}=rank\{p_{i,g},i=1,\cdots,N_{d}\}$denotes the rank value of $p_{i,g}$ over samples. Similarly, we can calculate the average rank of the proportion of protective SNPs for each normal control as follows:

$t_{j}=\sum_{g=1}^{G} t_{j,g}/G,j=1,\cdots,N_{c}$,

where $t_{j,g}=rank\{q_{j,g},j=1,\cdots,N_{c}\}$denotes the rank value of $q_{j,g}$over samples. Let $\tilde{s}_{i}=rank\{s_{1},\cdots,s_{N_{d}}\}$and $\tilde{t}_{j}=rank\{t_{1},\cdots,t_{N_{c}}\}$, where high values of $s_{i}$ and $t_{j}$will be assigned as high rank values. Then we identify cases and controls with the high rank values, respectively.

- **The third method:** We calculate the average of the weighted proportions of risky SNPs over all identified genes for each patient as follows:

$u_{i}=\sum_{g=1}^{G} w_{g}\cdot p_{i,g}/G,i=1,\cdots,N_{d}$,

where $w_{g}$is –log_10_(p-value) of a gene-based association test for the *g*th gene. Let $u_{i}^{*}=rank\{u_{i},i=1,\cdots,N_{d}\}$ denote the rank value of $u_{i}$over samples. If multiple gene-based association tests are performed, the average of over different tests is calculated. Similarly, we can calculate the average of the weighted proportions of protective SNPs over all identified genes for each normal control as follows:

$v_{j}=\sum_{g=1}^{G} w_{g}\cdot q_{j,g}/G,j=1,\cdots,N_{c}$.

Let $v_{j}^{*}=rank\{v_{j},j=1,\cdots,N_{c}\}$ denote the rank value of $v_{j}$over samples. Let $\tilde{u}_{i}=rank\{u_{1},\cdots,u_{N_{d}}\}$and$\tilde{v}_{j}=rank\{v_{1},\cdots,v_{N_{c}}\}$, where high values of $u_{i}$ and $v_{j}$will be assigned as high rank values. Then we identify cases and controls with the high rank values, respectively.

# We identify the respective hypertensive patients carrying rich risky SNPs (i.e., patients with a high rank value,$\tilde{p}_{i}$,$\tilde{s}_{i}$or $\tilde{u}_{i}$) and normotensive controls carrying rich protective SNPs (i.e., normal controls with a value of$\tilde{q}_{j}$,$\tilde{t}_{j}$or $\tilde{v}_{j}$) by using the three aforementioned methods. Next, we furthermore incorporate the case-control matching information into the selection of extremely discordant case-control pairs. We set thresholds of rank value, $\theta_{d}$and $\theta_{c}$, for selection of samples in disease and control groups. For each of the three aforementioned methods, we list all patients and controls that their rank values exceed the threshold. Ranks of risky scores in hypertensive patients and ranks of protective scores in normotensive controls are shown in Figure S3 below. In our study, when $\theta_{d}=\theta_{c}=350/400=0.875$ (i.e., top rank 50), we identified 12 patients and their corresponding matched controls that their rank values from all the three methods exceed the threshold (see Figure S3).

**Figure S3.** **Ranks of risky scores in hypertensive patients and ranks of protective scores in normotensive controls.** Ranks of risky/protective scores of hypertensive/normotensive samples were calculated by using three methods described above. A red point indicates a rank value of a sample. For the same sample, the ranks from the three methods are connected by a vertical line. All three rank values of a sample exceed the threshold of top rank 50 are presented in dark red points and a black connected line; their IDs are shown in the top panel. Otherwise, the rank values are indicated by light red points and a light blue connected line. (A) Ranks of risky scores in hypertensive patients. (B) Ranks of protective score in normotensive controls.

(A)


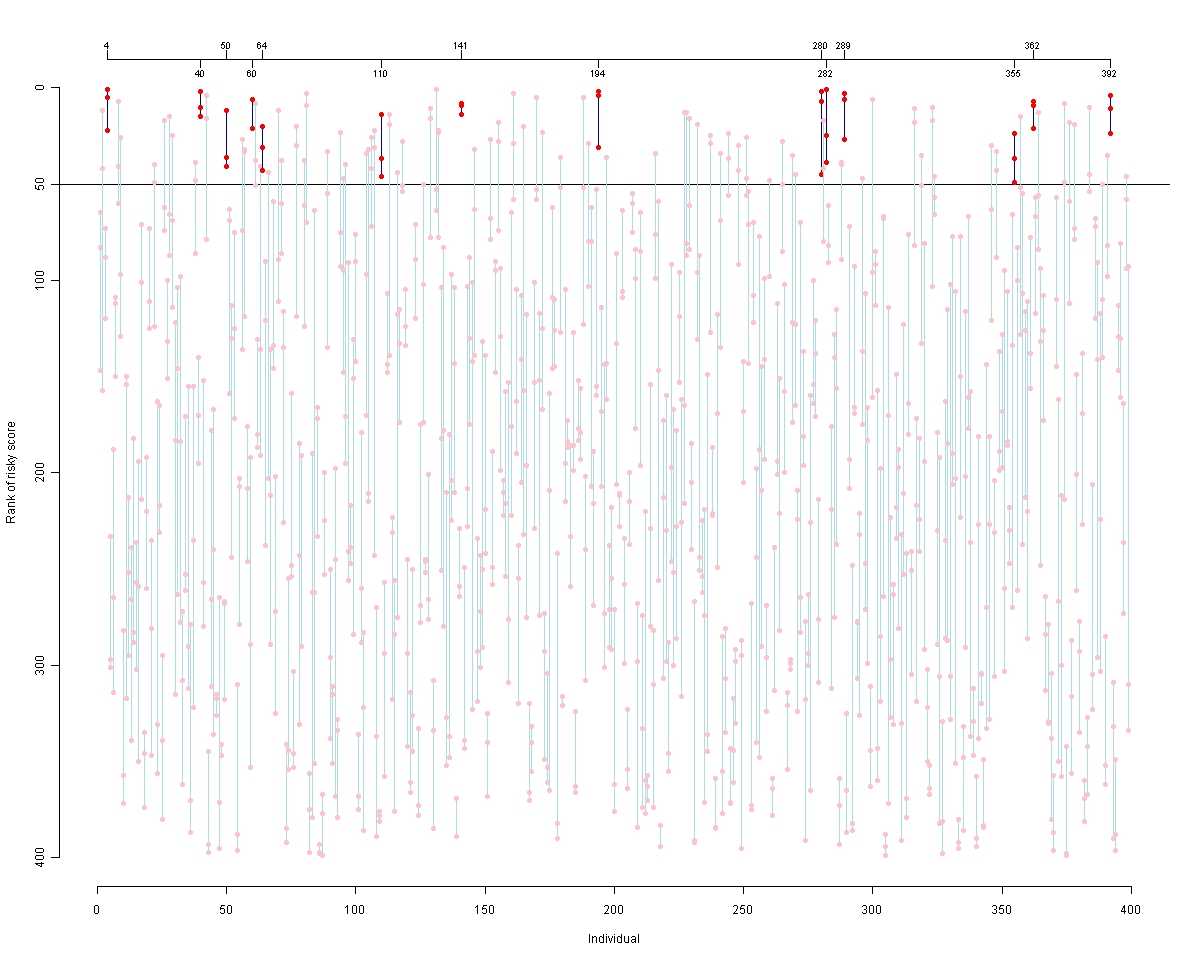


(B)


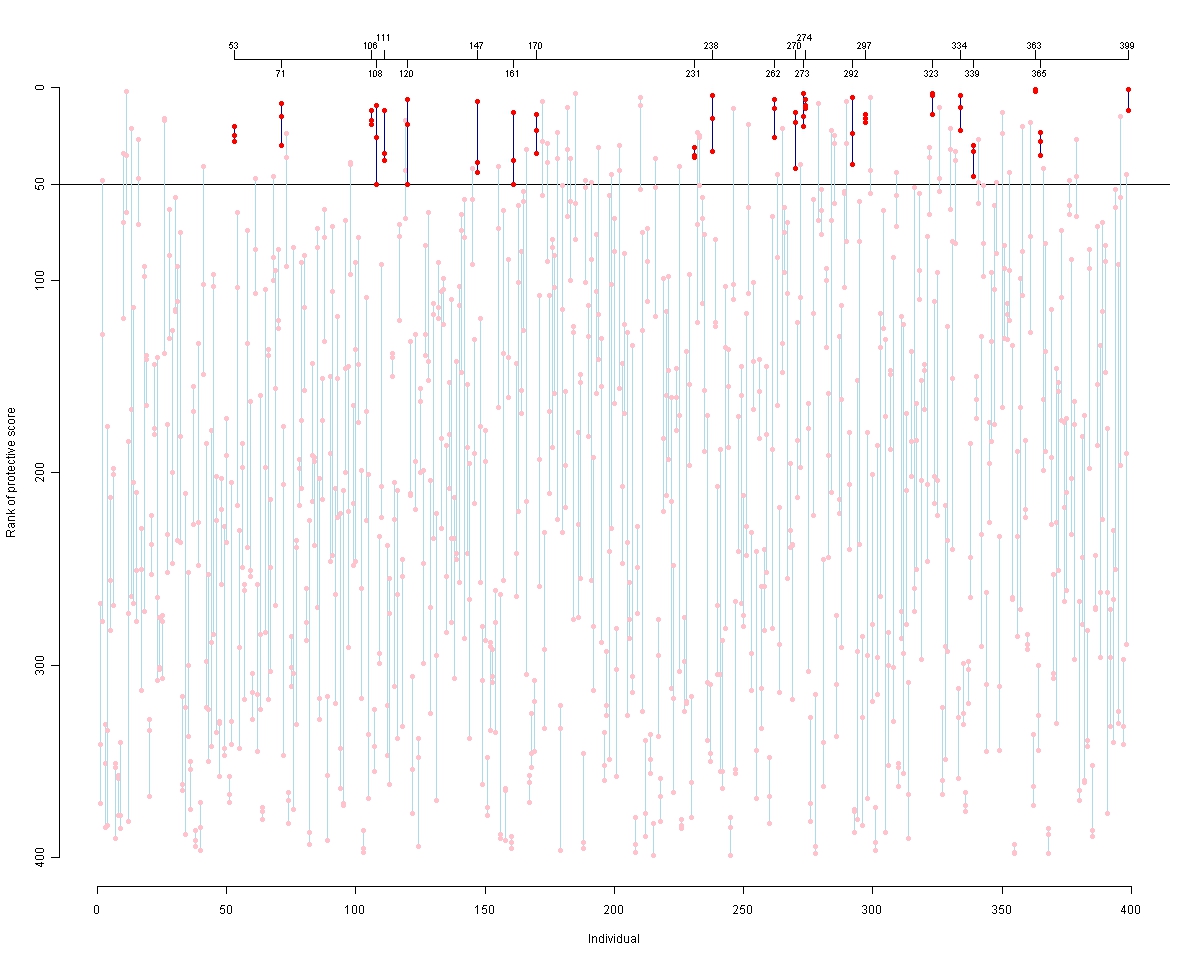

Supplement: Method S2 — Selection of extremely discordant case-control pairs. (DOC) [file pone.0032907.s009.doc]
